# The sigma-1 receptor modulates methamphetamine dysregulation of dopamine neurotransmission

**DOI:** 10.1038/s41467-017-02087-x

**Published:** 2017-12-20

**Authors:** Danielle O. Sambo, Min Lin, Anthony Owens, Joseph J. Lebowitz, Ben Richardson, Darin A. Jagnarine, Madhur Shetty, Meghan Rodriquez, Taiwo Alonge, Mishaal Ali, Jonathan Katz, Long Yan, Marcelo Febo, L. Keith Henry, Adriaan W. Bruijnzeel, Lynette Daws, Habibeh Khoshbouei

**Affiliations:** 1 Department of Neuroscience, University of Florida, Gainesville, FL 32611 USA; 2 Department of Cellular & Integrative Physiology, University of Texas Health Science Center at San Antonio, San Antonio, TX 78229 USA; 3 Department of Psychiatry, University of Florida, Gainesville, FL 32611 USA; 4 Department of Biomedical Sciences, University of North Dakota, Grand Forks, ND 58203 USA; 5 Psychobiology Section, Intramural Research Program, National Institute on Drug Abuse, National Institutes of Health, Baltimore, MD 21224 USA; 6 Max Plank Institute for Neuroscience Jupiter, Jupiter, FL 33458 USA

## Abstract

Dopamine neurotransmission is highly dysregulated by the psychostimulant methamphetamine, a substrate for the dopamine transporter (DAT). Through interactions with DAT, methamphetamine increases extracellular dopamine levels in the brain, leading to its rewarding and addictive properties. Methamphetamine also interacts with the sigma-1 receptor (σ_1_R), an inter-organelle signaling modulator. Using complementary strategies, we identified a novel mechanism for σ_1_R regulation of dopamine neurotransmission in response to methamphetamine. We found that σ_1_R activation prevents methamphetamine-induced, DAT-mediated increases in firing activity of dopamine neurons. In vitro and in vivo amperometric measurements revealed that σ_1_R activation decreases methamphetamine-stimulated dopamine efflux without affecting basal dopamine neurotransmission. Consistent with these findings, σ_1_R activation decreases methamphetamine-induced locomotion, motivated behavior, and enhancement of brain reward function. Notably, we revealed that the σ_1_R interacts with DAT at or near the plasma membrane and decreases methamphetamine-induced Ca^2+^ signaling, providing potential mechanisms. Broadly, these data provide evidence for σ_1_R regulation of dopamine neurotransmission and support the σ_1_R as a putative target for the treatment of methamphetamine addiction.

## Abstract (web summary)

The dopamine transporter (DAT), a regulator of dopamine homeostasis in the brain, and sigma-1 receptor (σ_1_R), an endoplasmic reticulum membrane protein, are both implicated in drug addiction. In this work, the authors investigate how σ_1_R modulates DAT response to methamphetamine.

## Introduction

The dopamine transporter (DAT) is a transmembrane protein implicated in multiple physiological and pathological conditions, including movement, reward, and drug addiction^1^. DAT facilitates the uptake of dopamine from the extracellular fluid back into the neuron and modulates the excitability of dopaminergic neurons, making it a crucial regulator of dopamine homeostasis in the brain^1,2^. DAT is also the primary target for the psychostimulants cocaine and methamphetamine (METH)^3,4^. The reinforcing effects and abuse potential of these psychostimulants directly correlate with their ability to increase extracellular dopamine levels in brain, albeit via different mechanisms^3^. Whereas cocaine increases extracellular dopamine by blocking DAT, METH increases extracellular dopamine levels by at least three well-described mechanisms: (1) competitive inhibition of dopamine uptake, (2) stimulation of dopamine efflux, and (3) internalization of DAT from the plasma membrane^5–8^. Furthermore, METH also increases the excitability of dopaminergic neurons in a DAT-dependent fashion^2,9,10^. The net result of these actions is a robust increase in extracellular dopamine levels in brain. Currently, there are no effective pharmacotherapies for the treatment of METH addiction.

A potential molecular target for the treatment of psychostimulant addiction is the sigma-1 receptor (σ_1_R). The σ_1_R is an endoplasmic reticulum (ER) chaperone protein widely expressed throughout brain including the midbrain and striatum, regions with high DAT expression^11^. Although two isoforms of the σR exist, σ_1_R and σ_2_R, the σ_1_R is more extensively investigated. Only recently was the σ_2_R cloned and identified as TMEM97, an ER membrane protein linked to cholesterol homeostasis and potentially other cellular functions^12,13^. In contrast, the σ_1_R was cloned over two decades ago, and its crystal structure was identified in 2016^26,27^, and there are currently more pharmacological and genetic tools to study the σ_1_R compared to the σ_2_R^14,15^. The σ_1_R acts as an intra-organelle cellular modulator, influencing signaling pathways, Ca^2+^ homeostasis, and ion channel activity^16^. The cellular localization of this protein is dynamic in nature. At rest, the σ_1_R is associated with another ER chaperone protein, binding immunoglobulin protein (BiP)^17^. Upon either ligand binding or an increase in intracellular Ca^2+^, the σ_1_R dissociates from BiP and translocates to other cellular compartments, including the plasma membrane^17,18^. Studies have shown that the σ_1_R is located in close contact with the plasma membrane at areas called subsurface cisternae^19,20^. These ER structures provide direct communication between the ER and plasma membrane for Ca^2+^ signaling and/or protein trafficking^21^. Although the mechanisms by which the σ_1_R translocates to the plasma membrane remain elusive, the σ_1_R has been shown to interact with and modulate the activity of various membrane proteins^16,22^, including recent evidence for interactions between the σ_1_R and DAT^23^.

σRs have been widely implicated in METH addiction. METH interacts with the σ_1_R at physiologically relevant concentrations^24^. Additionally, METH exposure increases σ_1_R levels in DAT-expressing brain regions of rodents, including the ventral tegmental area (VTA) and substantia nigra^11^. Given evidence that σR ligands attenuate METH-induced behavioral responses, hyperthermia, and neurotoxicity^24–29^, the increase in σ_1_R levels following METH exposure potentially serves as an compensatory mechanism to decrease the effects of METH. However, the mechanisms by which the σ_1_R regulates the METH-stimulated responses are largely unknown. In contrast to METH, the potential molecular mechanisms by which the σ_1_R modulates responses to cocaine have been more thoroughly examined^23,30–32^. It should be noted that while behavioral responses to both METH and cocaine are attributed to increased extracellular dopamine, METH rapidly enters the cell, releases dopamine from vesicular stores, and induces dopamine efflux via DAT^33,34^. In contrast, cocaine blocks uptake of dopamine via DAT. These mechanistic differences may result in distinct pharmacological manipulation of the cellular responses to METH compared to cocaine and distinct modulation of these responses via the σ_1_R. As such, the present study is focused specifically on σ_1_R modulation of METH dysregulation of dopaminergic neurons.

Our findings reveal that administration of the selective σ_1_R agonist PRE-084 prevents METH-stimulated, DAT-mediated increases in excitability of dopamine neurons, both in cultured dopamine neurons and dopamine neurons of midbrain slices. Furthermore, σ_1_R activation significantly reduces METH-stimulated, DAT-mediated dopamine efflux in vitro and in vivo, without affecting substrate uptake or DAT levels at the plasma membrane. Importantly, pretreatment with the σ_1_R agonist PRE-084 decreases METH-induced psychomotor responses, drug-seeking behavior, and enhancement of brain reward function; supporting the interpretation that σ_1_R activation decreases METH-mediated dopamine neurotransmission. Studies examining the potential functional mechanisms of these responses confirmed that the σ_1_R associates with DAT at or near the plasma membrane and that this association is potentiated after combined exposure to PRE-084 and METH. Consistent with previous reports that the σ_1_R regulates intracellular Ca^2+^ dynamics^17,35^, we also found that σ_1_R activation reduces METH-mediated increases in intracellular Ca^2+^, which is critical for METH-induced, DAT-mediated dopamine efflux^36^. Overall, these results suggest that activation of the σ_1_R reduces METH-stimulated DAT activity, thereby reducing METH-induced stimulation of dopaminergic neurotransmission and decreasing behavioral responses to METH. These responses are potentially mediated via a DAT-dependent mechanism and σ_1_R-modulation of intracellular Ca^2+^ homeostasis. These findings support the σ_1_R as a potential therapeutic target for the treatment of METH addiction.

## Results

### σ_1_R upregulation inhibits METH-stimulated firing activity

**Fig. 1:**
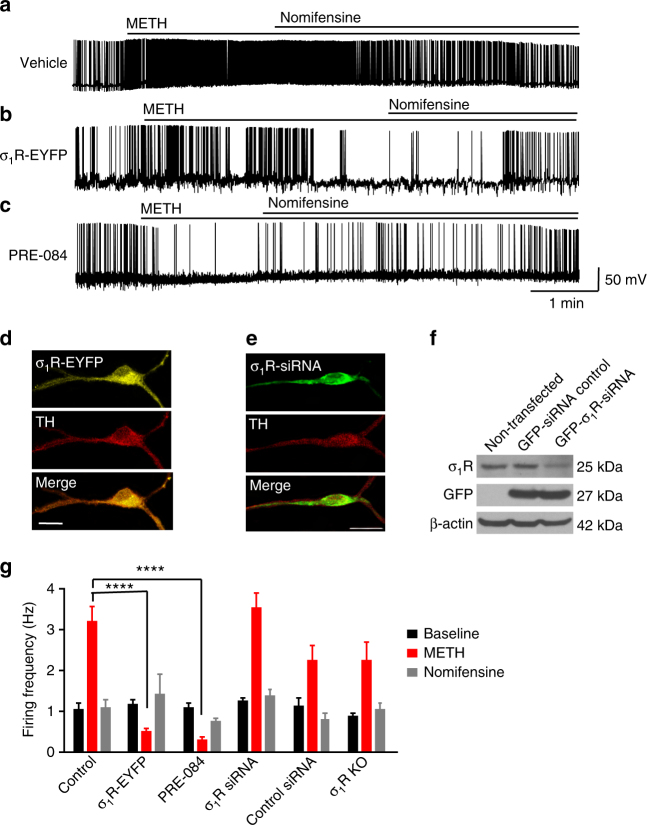
σ_1_R agonist or σ_1_R overexpression inhibits METH-induced, DAT-mediated increases in the firing rate of dopaminergic neurons. The spontaneous firing activity of mouse midbrain dopaminergic neurons was recorded at resting membrane potential in current clamp mode. Representative traces are shown. **a** Application of METH (10 μM) significantly increased the spontaneous firing rate frequencies above baseline levels (*n* = 11 neurons). The firing rate returned to baseline levels following application of the DAT blocker nomifensine (10 μM). **b** Overexpression of σ_1_R-EYFP (*n* = 10 neurons) or **c** pretreatment with 10 μM of the σ_1_R agonist PRE-084 (*n* = 12 neurons) blocked the METH-induced increase in firing rate of dopaminergic neurons. Similar to experiments in (a), application of nomifensine returned the firing rate to baseline levels in σ_1_R-EYFP expressing and PRE-084-treated neurons. **d** Representative image of a dopamine neuron expressing σ_1_R-EYFP immunolabeled for tyrosine hydroxylase (TH) (scale bar: 20 μm). **e** Representative image of a dopamine neuron expressing GFP-σ_1_R-siRNA immunolabeled for TH (scale bar: 20 μm). **f** Western blot shows σ_1_R levels were decreased after GFP-σ_1_R-siRNA treatment and unchanged in non-transfected or control siRNA treated cells. **g** Bar graph shows spontaneous firing rate (spikes per second) in different treatment groups at baseline, after METH treatment, and after subsequent nomifensine treatment. Dopamine neurons treated either with GFP-σ_1_R-siRNA (*n* = 10 neurons) or with control siRNA (*n* = 5 neurons) and neurons derived from σ_1_R knockout mice (*n* = 8 neurons) had no change in the METH-induced increase in firing rate compared to control. (*F*_(10,96)_ = 14.2, *P* < 0.0001, two-way ANOVA; Sidak’s test for multiple comparisons within baseline, METH, and nomifensine; **** *P* < 0.001). Data is represented as mean ± SEM

To investigate the influence of σ_1_Rs on METH-stimulated, DAT-mediated activity, we first examined whether σ_1_R activation or overexpression influences DAT-dependent, METH-stimulated spontaneous firing activity of dopaminergic neurons. As previously reported^2,9,10^, dopamine neurons were whole-cell patch clamped and spontaneous firing rates were recorded in the continued presence of the D_1_ receptor antagonist SCH23390 (5 μM) and the D_2_ receptor antagonist sulpiride (5 μM) in order to isolate DAT-dependent changes in firing activity. As shown previously^9,10^, administration of METH (10 μM) significantly increased the firing rate of dopamine neurons, which returned to baseline after application of the DAT blocker nomifensine (10 μM) (Fig. 1a), suggesting that the increase in firing activity is DAT-dependent. To assess whether σ_1_R activation or overexpression influences METH-stimulated, DAT-mediated excitability of dopamine neurons, firing activity was recorded in neurons overexpressing an enhanced yellow fluorescent protein (EYFP)-tagged σ_1_R (σ_1_R-EYFP) (Fig. 1d) or neurons pretreated with the selective σ_1_R agonist PRE-084 (10 μM, IC_50_ = 44 nM or *K*_i_ 2.2 nM)^37^. Whereas neither σ_1_R-EYFP overexpression (Fig. 1b) nor PRE-084 pretreatment (Fig. 1c) affected baseline firing activity, both manipulations inhibited METH-stimulated firing activity to levels below the basal firing rate (Fig. 1g). Similar to control, treatment with nomifensine brought the firing rate back to baseline. Unlike σ_1_R upregulation, we found that treatment with green fluorescent protein (GFP)-tagged σ_1_R-siRNA (Fig. 1e) or a GFP-tagged-scrambled RNA sequence did not alter basal or METH-induced firing activity of dopamine neurons (Fig. 1g). As shown in Fig. 1f, σ_1_R-siRNA decreased σ_1_R expression, whereas the control siRNA had no effect. Like siRNA treatment, dopamine neurons generated from σ_1_R knockout mice showed no change in basal or METH-mediated firing activity (Fig. 1g). Taken together, these results suggest that whereas downregulation of σ_1_R expression had no effect, σ_1_R upregulation decreases METH-stimulation of dopamine neurotransmission.

**Fig. 2:**
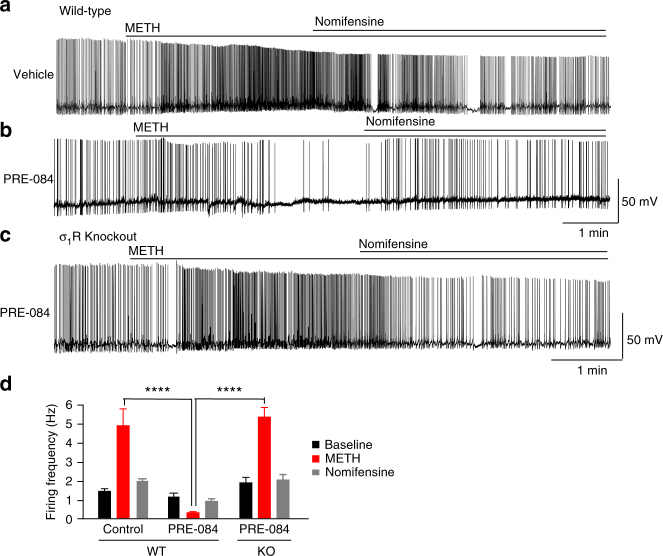
σ_1_R activation decreases METH-stimulated firing activity in dopaminergic neurons of midbrain slices in wild-type but not σ_1_R knockout mice. Slice recordings were performed in ventral tegmental area slices derived from 4 to 6-week-old mice. **a** Representative trace of spontaneous firing activity reveals METH-mediated (10 μM) increases firing activity that are blocked with application of nomifensine (10 μM) (*n* = 8 neurons). **b** While baseline activity was unaffected, 30-min pretreatment with 10 μM PRE-084 blocked the ability of METH to increase the firing activity in the dopamine neurons. Application of nomifensine returned the firing rate to baseline (*n* = 9 neurons). **c** 30-min pretreatment with 10 μM PRE-084 in σ_1_R knockout mice resulted in no change in the METH-induced increase in firing activity, supporting the conclusion that the effect of PRE-084 on METH-stimulated firing activity is σ_1_R-dependent (*n* = 8 neurons). **d** Bar graph shows the spontaneous firing rate (spikes per second) in different treatment groups at baseline, after METH treatment, and after subsequent nomifensine treatment (*F*_(4,50)_ = 19.52, *P* < 0.0001, two-way ANOVA; Tukey’s test for multiple comparisons within baseline, METH, and nomifensine; ****P < 0.001). Data is represented as mean ± SEM

Considering the possibility that σ_1_Rs may differentially regulate METH-stimulated responses in the neuronal cultures generated from postnatal mice compared to adults, we repeated electrophysiological recordings in the VTA of acutely prepared adult mouse brain slices. Consistent with our findings from cultured dopamine neurons and previous studies^2,9,10,38^, METH increased the firing frequency of dopamine neurons in midbrain slices via a DAT-dependent mechanism (Fig. 2a) and pretreatment with PRE-084 (10 μM) again dramatically inhibited the firing activity of neurons in response to METH (Fig. 2b). To further examine the specificity of PRE-084 for σ_1_Rs, recordings were performed in slices from σ_1_R knockout mice. Supporting the σ_1_R-dependency for the actions of PRE-084, pretreatment with the agonist had no effect on basal or METH-induced firing activity in slices prepared from mice lacking the σ_1_R (Fig. 2c). Average firing frequencies across the different groups is shown in Fig. 2d. Overall, these findings support activation of σ_1_Rs as a mechanism to suppress METH-induced enhancement of dopamine cell firing.

### σ_1_R activation decreases METH-stimulated DA efflux

**Fig. 3:**
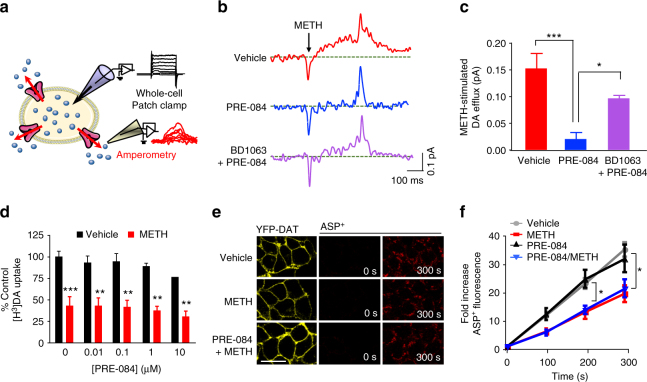
σ_1_R activation decreases METH-stimulated dopamine efflux without affecting uptake. **a** Schematic of the setup is shown. YFP-DAT cells loaded with dopamine were voltage clamped while oxidative currents were measured via an amperometric electrode. Dopamine is represented in purple and DAT in pink. Cell, molecules, and proteins images are courtesy of Servier Medical Art, licensed under CC BY 3.0 (https://creativecommons.org/licenses/by/3.0/legalcode). **b** Representative traces show METH-stimulated (10 µM) dopamine efflux after 30-min treatment with vehicle, 1 µM PRE-084, or 1 µM BD1063 plus 1 µM PRE-084. METH increased dopamine efflux (top panel), while PRE-084 significantly decreased the effect of METH (middle panel). BD1063 blunted the effect of PRE-084. **c** Bar graph shows the average METH-stimulated dopamine efflux above baseline following treatment with vehicle, PRE-084, or BD-1063 + PRE-084 (*n* = 4, 4, and 6 cells, respectively, *F*_(2,11)_ = 15.18, *P* = 0.0007, one way ANOVA; Tukey’s test for multiple comparisons, *P < 0.05 and *** *P* < 0.001). **d** [^3^H]DA uptake was measured in FLAG-DAT cells. 10 μM METH (15 min) significantly decreased [^3^H]DA uptake compared to vehicle. 30-min PRE-084 treatment had no effect on basal uptake or METH-inhibition of uptake. Data is represented as percent vehicle control (*n* = 3 independent experiments; *F*_(1,20)_ = 90.34, *P* = 0.0009, two-way ANOVA for Vehicle × METH; *F*_(4,20)_ = 1.363, *P* = 0.2821, two-way ANOVA for PRE-084 treatment; Bonferroni’s test for multiple comparisons of Vehicle vs. METH, **P < 0.01 and ***P < 0.001). **e** ASP^+^ uptake was measured in YFP-DAT cells, shown in left panels (scale bar: 10 μm). **f** Line graph shows average fold ASP^+^ fluorescence increase over time. METH (*n* = 11) significantly decreased ASP^+^ uptake compared to vehicle (*n* = 14), while PRE-084 treatment did not affect baseline (*n* = 9) or METH-inhibition of uptake (*n* = 8) (*n* represents independent experiments; *F*_(3,63)_ = 156.8, *P* < 0.0001, two-way ANOVA for Time; *F*_(9,117)_ = 4.959, *P* = 0.0480, two-way ANOVA for Treatment; Bonferroni’s test for multiple comparisons, *P < 0.05 for METH groups vs. non-METH groups at 300 s). Data is represented as mean ± SEM

METH stimulates DAT-mediated dopamine efflux by reverse transport of dopamine, the primary mechanism for METH-stimulated dopamine release in brain^39^. To explore whether σ_1_R activation attenuates METH-stimulated, DAT-mediated dopamine efflux, we used simultaneous patch-clamp electrophysiology and amperometry in DAT-expressing cells. The experimental set up is shown in Fig. 3a. Consistent with previous reports^40^, application of METH (10 µM) resulted in a voltage-dependent increase in dopamine efflux measured as an increase in oxidative current (Fig. 3b, top panel). Remarkably, pretreatment with the σ_1_R agonist PRE-084 (1 µM) significantly reduced METH-stimulated dopamine efflux without affecting baseline dopamine efflux (Fig. 3b, middle panel). Pretreatment with the σ_1_R antagonist BD1063 (1 µM) significantly decreased (~60%) the ability of the σ_1_R agonist to inhibit METH-stimulated dopamine efflux (Fig. 3b, c). Consistent with σ_1_R-inhibition of METH-stimulated neuronal activity in dopamine neurons, these data support the interpretation that activation of σ_1_Rs blunts METH-stimulation of dopaminergic neurotransmission.

### σ_1_R activation does not affect DA uptake

We next examined whether the σ_1_R agonist PRE-084 effects basal or METH-inhibition of substrate uptake. DAT-mediated [^3^H]-dopamine ([^3^H]DA) uptake was measured in DAT expressing cells, and nonspecific uptake was measured in the presence of nomifensine. The concentration of unlabeled dopamine used with [^3^H]DA was determined in Supplementary Fig. 1a. As predicted, METH (10 µM) significantly reduced [^3^H]DA uptake (Fig. 3d). Unlike its effect on dopamine efflux, PRE-084 pretreatment had no effect on baseline [^3^H]DA uptake or METH-inhibition of [^3^H]DA uptake (Fig. 3d). Similar to the lack of effect of σ_1_R knockdown or knockout in Fig. 1, the σ_1_R antagonist BD1063 also did not affect baseline or METH-inhibition of [^3^H]DA uptake (Supplementary Fig. 1b). Considering the possibility that σ_1_R-regulation of dopamine uptake is beyond the temporal resolution of this radiometric assay and the possibility that decreased dopamine efflux might influence net [^3^H]DA uptake measured, we measured uptake of a fluorescent substrate of DAT, 4-(4-dimethylamino-styryl)-*N*-methylpyridinium (ASP^+^), via live cell confocal microscopy in YFP-DAT cells. Unlike [^3^H]DA uptake, ASP^+^ uptake can provide millisecond time resolution and allows for the measurement of substrate uptake without the influence of substrate efflux^41^. As shown in Fig. 3e, METH treatment (10 µM) decreased ASP^+^ uptake in YFP-DAT cells over time. Similar to uptake results measured via radiometric assay (Fig. 3d), PRE-084 treatment did not affect either baseline or METH-inhibition of substrate uptake at any time point examined (Fig. 3f). Therefore, while PRE-084-activation of σ_1_R significantly decreased METH-stimulated, DAT-mediated dopamine efflux, it had no effect on the forward substrate transport via DAT.

### σ_1_R activation decreases METH-stimulated DA release in vivo

**Fig. 4:**
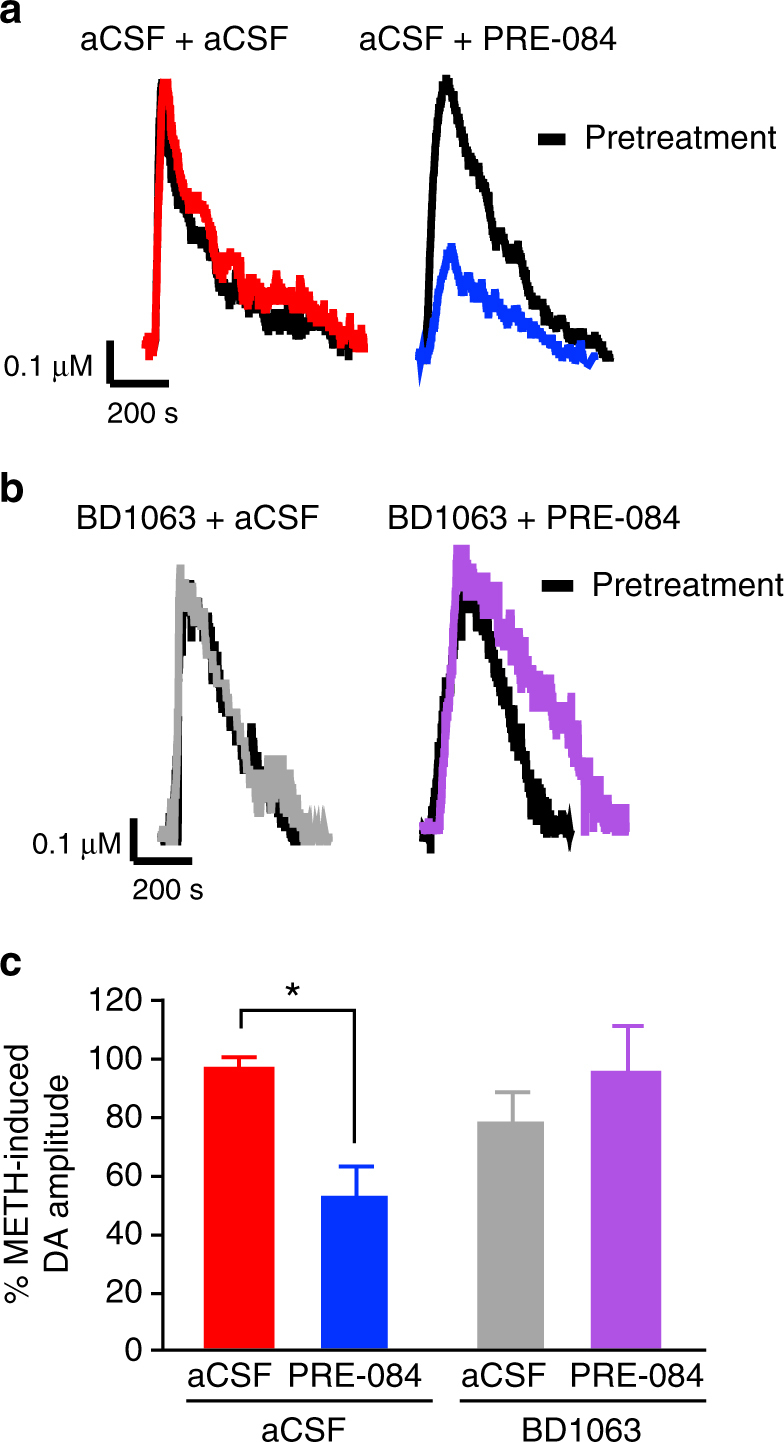
In vivo σ_1_R activation in the dorsal striatum inhibits METH-induced dopamine efflux. METH was locally applied into the dorsal striatum of anesthetized mice. Representative oxidation currents (converted to a micromolar concentration using a calibration factor determined in vitro) are shown in (**a**) and (**b**). METH signals produced after pretreatment are overlaid with signals obtained after the secondary treatment for ease of comparison. **a** Representative oxidation traces produced by METH-induced dopamine efflux after pretreatment aCSF treatment (black lines) or subsequent aCSF (red line) or PRE-084 (blue line) treatment. **b** Representative oxidation currents produced by METH-induced dopamine efflux after BD1063 pretreatment (black lines) or subsequent aCSF (gray line) or PRE-084 (purple line) treatment. **c** Comparison of the signal amplitude percent change over baseline (pre-aCSF or pre-PRE-084) reveals that intrastriatal application of PRE-084 (*n* = 9) but not aCSF (*n* = 6) significantly decreased the amplitude of dopamine release. Application of BD1063 prior to PRE-084 prevented this effect (*n* = 4 BD1063 + aCSF, *n* = 6 for BD1063 + PRE-084, *F*_(1,21)_ = 6.06, *P* = 0.0226, Sidak’s test for multiple comparisons, *P = 0.0222). Data is represented as mean ± SEM

To highlight the biological significance of these cellular events, we next examined the effect of σ_1_R activation on METH-stimulated dopamine release in vivo using high-speed chronoamperometry in the dorsal striatum of anesthetized adult C57BL/6J mice. As expected, microinjection of METH (80 pmol) into the dorsal striatum caused robust dopamine release. Compared to artificial cerebral spinal fluid (aCSF) pretreatment, intrastriatal application of PRE-084 (10 pmol) 15 min prior to METH blunted METH-evoked dopamine release by ~50% (Fig. 4a), recapitulating our findings in vitro. Administration of BD1063 (10 pmol) prior to PRE-084 blocked the effect of PRE-084 (Fig. 4b), supporting the selectivity of this effect for the σ_1_R. BD1063 had no effect on METH-stimulated dopamine release by itself. The percent increase in dopamine release after METH is shown in Fig. 4c. Pretreatment with either σ_1_R ligand had no effect on rise time, release, or clearance rate of the dopamine signal following METH treatment (Supplementary Fig. 2a, b). Similarly, pretreatment with PRE-084 or BD1063 did not influence the ratio of currents produced by reduction and oxidation (red:ox), which provides a signature of the neurotransmitter being recorded (Supplementary Fig. 2c). Overall, these data further support the interpretation that activation of σ_1_Rs blunts METH-stimulation of dopamine neurotransmission in the brain.

### σ_1_R activation decreases METH-stimulated locomotor responses

**Fig. 5:**
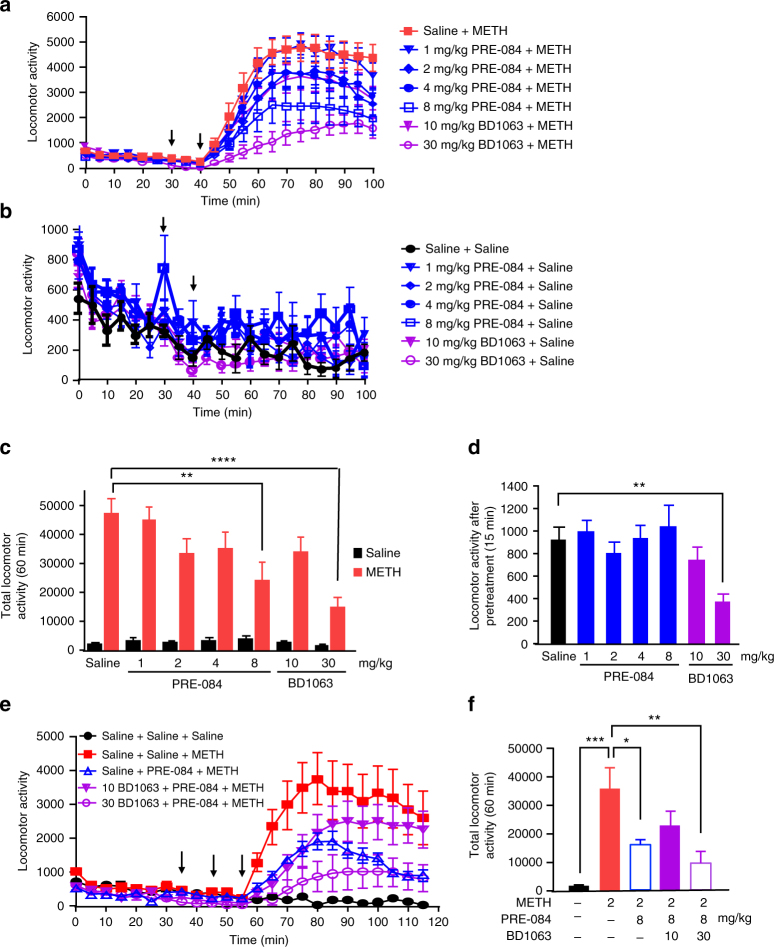
σ_1_R activation decreases acute METH-mediated behavioral responses. After 30-min habituation to the locomotor chamber, C57BL/6 mice were pretreated with either saline, 1–8 mg/kg PRE-084, or 10 or 30 mg/kg BD1063 by i.p. injection followed by treatment with (**a**) 2 mg/kg METH or (**b**) saline after 15 min. Locomotor activity was detected via automated locomotor chambers during the habituation, pretreatment, and 60 min after saline or METH treatment at 5 min bins. **c** Graph shows the total locomotor activity after the second injection (METH or saline). Whereas METH alone increased locomotion, 8 mg/kg PRE-084 and 30 mg/kg BD-1063 both decreased METH-stimulation of locomotion, while all other doses tested had no effect on baseline or METH-stimulated locomotion. (*n* = 8 mice for METH treatment, 6 mice for saline treatment, *F*_(6,86)_ = 3.418, *P* = 0.0045 two-way ANOVA, Sidak’s multiple comparisons within saline or METH, ***P = 0.0007 and ****P < 0.0001). **d** Locomotor activity measured during the 15 min after pretreatment but prior to METH treatment in experiments performed in Fig. 5a and b revealed 30 mg/kg BD1063 acutely decreased locomotor activity in the rodents. (*n* = 8 mice for all groups, *F*_(6,93)_ = 3.356, *P* = 0.0049, one-way ANOVA; Dunnett’s test for multiple comparisons against saline, **P = 0.0095). **e** C57BL/6 mice were pretreated with either saline or 10 or 30 mg/kg BD1063 by i.p. injection followed by treatment with saline or 8 mg/kg PRE-084 after 15 min. Mice then received a third injection of either saline or 2 mg/kg METH after 15 min and locomotor activity was measured for 60 min. **f** While 10 mg/kg BD1063 modestly blocked of the effect of PRE-084 on reducing METH-stimulated locomotor activity over time, treatment with 30 mg/kg BD1063, similar to Fig. 5a and c, resulted in a significant decrease in METH-induced locomotor activity (*F*_(4,35)_ = 4.247, *P* = 0.0002, one-way ANOVA; Tukey’s test for multiple comparisons, ***P = 0.001, *P < 0.02, **P = 0.0034). Data is represented as mean ± SEM

Decreased METH-stimulated firing activity of dopamine neurons and dopamine release support the hypothesis that σ_1_R activation decreases a collection of behavioral responses to METH. Therefore, we first examined the effect of the σ_1_R agonist PRE-084 on METH-stimulated locomotion in C57BL/6J mice. After 30 min habituation to the locomotor chamber, animals were pretreated with saline, PRE-084, or BD1063 at different doses followed by treatment with saline or 2 mg/kg METH after 15 min. As previously reported^28^, 2 mg/kg METH elicited a marked locomotor response in rodents (Fig. 5a and c, red). While the σ_1_R agonist (1–8 mg/kg) alone did not alter basal locomotion (Fig. 5b and c), pretreatment with 8 mg/kg of PRE-084, as well as 30 mg/kg of BD1063, but not lower doses of either drugs, reduced METH-stimulated locomotor activity (Fig. 5a and c). Interestingly, when comparing locomotor activity during the pretreatment period before mice received METH or saline, mice receiving 30 mg/kg of BD1063 showed a significant decrease in activity compared to all other groups (Fig. 5d). Other studies have also shown potential sedative properties of σR antagonism^42,43^, suggest the ability of 30 mg/kg of BD1063 to blunt the locomotor effects of METH may be a sedative effect unrelated to dopamine neurotransmission.

The ability of both the σ_1_R agonist and antagonist to decrease METH-stimulated locomotor responses demonstrates the complexity of using combined σ_1_R ligands in vivo. To further examine the specificity of 8 mg/kg PRE-084 for the decrease in the METH-stimulated locomotor response, mice were pretreated with 10 or 30 mg/kg of BD-1063 prior to treatment with 8 mg/kg PRE-084 and METH (Fig. 5e). Pretreatment with 10 mg/kg of BD1063 produced a partial attenuation of the ability of PRE-084 to suppress METH-stimulated locomotor activity, particularly during the 30–60-min period following METH treatment (Fig. 5f). Unsurprisingly, considering its potential sedative effects and effects on METH-stimulated locomotor activity alone, 30 mg/kg BD1063 did not block the effect of PRE-084 (Fig. 5d). Overall, these studies support the notion that PRE-084 reduces METH-induced neurotransmission, while systemic presence of multiple drugs and their interactions on METH-induced psychomotor responses remain complex.

### σ_1_R activation reverses the reinforcing properties of METH

**Fig. 6:**
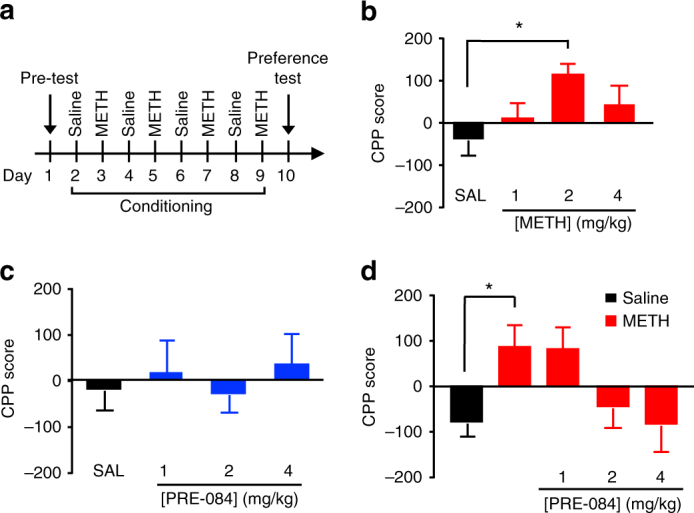
σ_1_R activation decreases METH-induced conditioned place preference. **a** Experimental paradigm for conditioned place preference (CPP) is shown. **b** Dose-response experiment using 1, 2, and 4 mg/kg METH (*n* = 11, 12, 11 C57BL/6 mice, respectively) produced reproducible CPP for METH. 2 mg/kg METH showed the largest CPP on the post-conditioning test compared to saline (SAL)-treated mice (*F*_(3,41)_ = 2.954, *P = *0.0436; with Tukey’s test for multiple comparisons, *P < 0.05). Therefore, 2 mg/kg METH was used for all further CPP studies. **c** Neither 1, 2, nor 4 mg/kg PRE-084 (*n = *4, 8, and 8 mice, respectively) showed CPP or conditioned place aversion compared to saline treatment (*n* = 8 mice). **d** Mice receiving 2 mg/kg METH (*n* = 12 mice) exhibited significant CPP compared to saline-treated animals (*n* = 12 mice). Treatment with 1, 2, and 4 mg/kg (*n* = 11 mice each) of PRE-084 before METH exposure decreased the CPP response, which was not significantly different from saline treatment (*F*_(4,52)_ = 3.695, *P* = 0.0101, one-way ANOVA; Bonferroni’s test for multiple comparisons, *P* = 0.0388). Data is represented as mean ± SEM

Although psychomotor responses reveal insight into the acute effects of METH, it does not address the rewarding properties of METH. Therefore, we examined the effect of the σ_1_R agonist PRE-084 on the acquisition of METH-induced conditioned place preference (CPP). CPP measures drug-associated contextual preference as an index of reward or aversion, in which a previously neutral environment paired with a rewarding drug, such as METH, acquires reinforcing properties^44^. The experimental design for the CPP paradigm used in this study is shown in Fig. 6a. Briefly, mice received either saline on one side of the CPP chamber or METH on the opposite side of the chamber on alternating days. The CPP score is measured on a final preference test day as the time spent on the drug-paired side minus time spent on the saline-paired side. Consistent with the literature, METH (2 mg/kg) induced CPP (Fig. 6b)^28^ and treatment with PRE-084 alone did not induce CPP or conditioned place aversion (Fig. 6c)^45^. Pretreatment with the σ_1_R agonist PRE-084 15 min before METH treatment during the conditioning phase dose-dependently reduced the acquisition of METH-induced CPP, to the extent that CPP scores of PRE-084 treated animals were not significantly different from animals treated with saline (Fig. 6d). These results suggest that σ_1_R activation not only attenuates METH-stimulated dopamine neurotransmission and acute psychomotor responses, it also reduces METH-induced drug-seeking behavior.

### σ_1_R activation diminishes the rewarding effects of METH

**Fig. 7:**
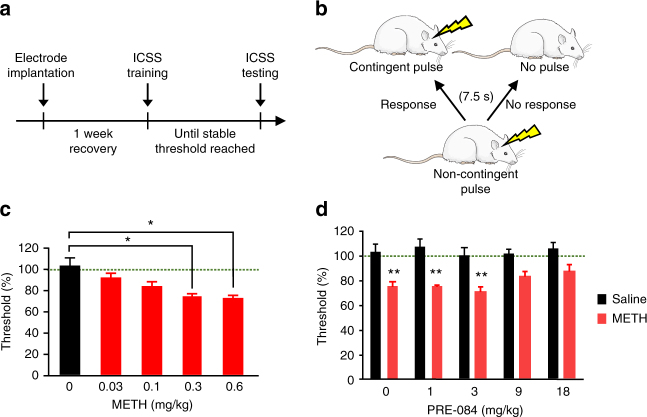
σ_1_R activation decreases the effects of METH on reward thresholds. **a** Experimental timeline for intracranial self-stimulation (ICSS) experiments is shown. **b** Schematic demonstrating ICSS testing is shown. Rodents receive a non-contingent pulse at a given frequency and are given 7.5 s to respond. Positive responding by the rodents results in a contingent pulse at the same frequency as the non-contingent pulse. The reward threshold is defined as the minimal frequency required to elicit a response. Rat image is courtesy of Servier Medical Art, licensed under CC BY 3.0 (https://creativecommons.org/licenses/by/3.0/legalcode). **c** The dose–response studies revealed METH dose-dependently lowers brain rewards thresholds in rats as measured via ICSS. Data is presented as percent vehicle control (*n* = 8 rats; *F*_(7, 28)_ = 3.479, *P* = 0.0083, repeated measures one-way ANOVA; Dunnett’s test for multiple comparisons against control, *P < 0.05). **d** PRE-084 treatment attenuated the METH-induced (0.3 mg/kg) decrease in brain reward thresholds (*n* = 6 saline rats, 8 METH rats; *F*_(1,12)_ = 75.23, *P* < 0.0001, two-way ANOVA; Bonferroni’s test for multiple comparisons, **P < 0.01 saline vs. METH; ^+^ *P* < 0.05 indicates a higher brain threshold compared to METH and 3 mg/kg of PRE-084). Data is represented as mean ± SEM

Beyond acute and reinforcing behavior, we utilized intracranial self-stimulation (ICSS) in rats to investigate whether σ_1_R agonist treatment attenuates METH’s potential for abuse indicated by increased self-stimulation^46^. ICSS is an operant paradigm where animals are trained to self-stimulate the brain via an electrode^47^. Acute administration of drugs of abuse lower the brain reward thresholds, measured as the minimal stimulation intensity required to maintain ICSS, indicative of a potentiation of brain reward function^48,49^. The METH doses and route of administration in this study were modeled after a previous report^50^. The experimental timeline is shown in Fig. 7a, and the paradigm for measuring threshold is shown in Fig. 7b. Similar to a recent report^50^, acute administration of METH lowered brain reward thresholds (Fig. 7c). Consistent with our in vitro and in vivo studies, the σ_1_R agonist PRE-084 attenuated METH-induced reduction of brain reward thresholds in a dose-dependent manner, such that PRE-084 pretreatment diminished the difference between brain reward thresholds of METH-treated rats and saline controls (Fig. 7d). The ICSS latency, or time between non-contingent pulse and response, was not affected (Supplementary Fig. 3). Notably, PRE-084 alone does not alter ICSS thresholds, consistent with CPP data in Fig. 6 that PRE-084 is neither rewarding nor aversive as drugs that induce aversion elevate ICSS thresholds. Collectively, these data support the hypothesis that σ_1_R activation reduces the rewarding effects of METH, without affecting basal dopamine neurotransmission and reward function or inducing aversive behavior.

### σ_1_R activation does not affect METH-mediated DAT trafficking

Since the availability of DAT at the plasma membrane correlates with its function, we investigated whether σ_1_R activation influences METH-stimulated trafficking of the transporter. Cell surface biotinylation was utilized to measure membrane levels of YFP-DAT in cells pretreated with vehicle, 0.1, or 1 µM PRE-084 followed by vehicle or 10 µM METH exposure. Total DAT and total protein levels remained constant after all drug treatments. Supplementary Fig. 4a shows representative immunoblots of biotinylated DAT (surface DAT) and total DAT. As predicted, METH treatment decreased DAT levels in the biotinylated fraction, whereas PRE-084 had no effect on basal or METH-stimulated cell surface redistribution of DAT (Supplementary Fig. 4b). Considering the possibility that σ_1_R activation accelerates the rate of DAT trafficking beyond detection by the biotinylation assay, we used live cell total internal reflection microscopy (TIRFM) to measure time-dependent DAT trafficking. Consistent with previous reports^51,52^, METH-induced DAT internalization measured as a decrease in the YFP-DAT fluorescence signal in the TIRF field (Supplementary Fig. 4c and d). PRE-084 pretreatment did not alter vehicle or METH-induced cell surface redistribution of DAT at any time points, confirming surface biotinylation data and suggesting that PRE-084 activation of σ_1_R does not increase or decrease basal DAT trafficking or METH-stimulated DAT internalization. Therefore, mechanisms other than altered DAT trafficking must underlie σ_1_R attenuation of METH activity on DAT.

### σ_1_R co-immunoprecipitates with DAT

**Fig. 8:**
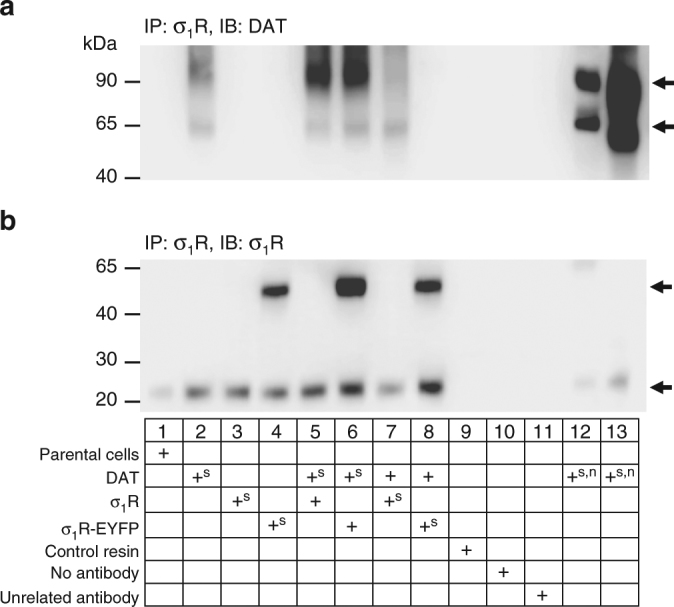
Co-immunoprecipitation reveals interaction between σ_1_R and DAT. **a** Western blots of immunoprecipitated (IP) HEK-293 extracts with an anti-σ_1_R antibody and probing with an anti-DAT antibody identifies bands corresponding to mature (top arrow) and immature forms (bottom arrow) of DAT only when σ_1_R and DAT are co-expressed. **b** Re-probing of the membrane with anti-σ_1_R shows expression of σ_1_R (lower arrow) and σ_1_R-EYFP (top band). The control resin, absence of specific antibody, and presence of a non-specific antibody (lanes 9–11) serve as negative controls. The σ_1_R band represents ectopic and endogenous σ_1_R expression. +^s^ indicates stable expression of the respective protein. +^n^ indicates samples from crude lysates (non-IP). Twenty micrograms of total protein was used in the lane 12 non-IP sample, whereas 40 μg was used in the lane 13 non-IP sample. The representative blots depict the findings from ≥3 independent experiments

Functional associations of DAT with other proteins regulates the activity of the transporter. Importantly, the σ_1_R interacts with and modulates the activity of various plasma membrane proteins^16,22^. Therefore, we considered the possibility that σ_1_R associates with DAT and thus regulates its function. Recently, the σ_1_R has been shown to associate with DAT and promote a more outward-facing confirmation of the transporter^23^. Confirming these findings, we found that immunoprecipitation of σ_1_Rs in cells co-expressing σ_1_R or σ_1_R-EYFP and DAT resulted in isolation of DAT (Fig. 8a), supporting the σ_1_R association with the transporter. Consistent with findings by Hong et al., both the immature (~65 kDa) and high molecular weight mature forms of DAT immunoprecipitated with σ_1_R, as indicated by the arrows (Fig. 8a). Co-immunoprecipitation of DAT with the σ_1_R occurred with both endogenous σ_1_R (lane 2), as well as overexpressed untagged or EYFP-tagged with σ_1_R (lanes 5 through 7). The ability of σ_1_R-EYFP to co-immunoprecipitate with DAT (lane 6) suggests that the C-terminal tag on the σ_1_R does not interfere with its ability to interact with the transporter. Interestingly, for reasons that remain unclear, cells stably expressing σ_1_R-EYFP (lane 8), but not transiently expressing σ_1_R-EYFP (lane 6), resulted in a dramatic decrease in DAT expression; therefore, we did not use this combination (stable σ_1_R-EYFP and transient DAT expression) for any further experiments. Confirmation of σ_1_R expression is shown in Fig. 8b where endogenous and transiently expressed σ_1_Rs are detected on subsequent Western blot using a second σ_1_R antibody. Neither protein was detected in non-transfected parental HEK cells (lane 1) or when the control resin, no antibody, or an unrelated antibody (*Drosophila* SERT) were used (lanes 9–11). DAT and σ_1_R expression in crude lysates is shown in Supplementary Fig. 5.

### σ_1_R is colocalized with DAT at the plasma membrane

**Fig. 9:**
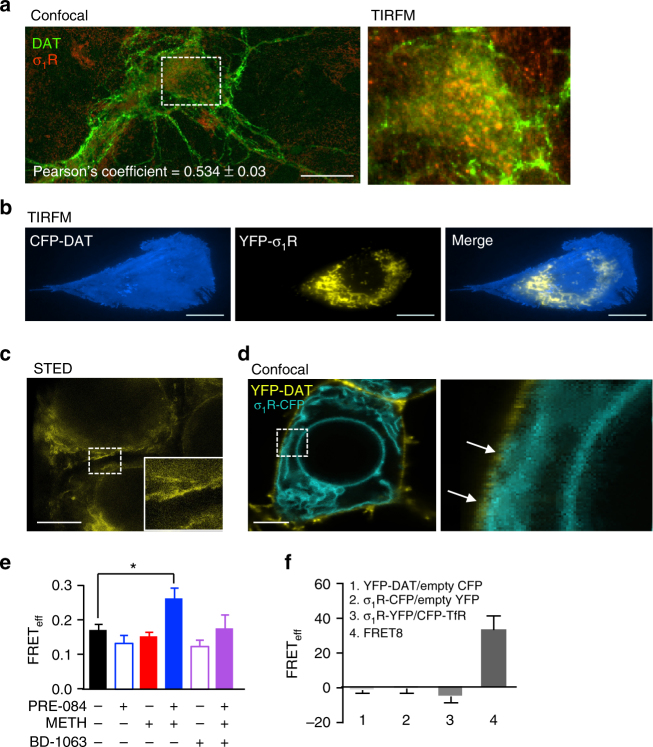
σ_1_R is co-localized with the dopamine transporter at or near the plasma membrane. **a** Representative confocal image of immunolabeled endogenous DAT (green) and endogenous σ_1_R (red) in primary culture of dopaminergic neurons shows DAT and σ_1_R are co-localized (shown in orange). The Pearson’s coefficient = 0.534 ± 0.03, *n* = 24 neurons (scale bar: 25 μm). Total internal reflection fluorescence microscopy (TIRFM) imaging of the neuronal cell body shows co-localization occurring at or near (<150 nm) the plasma membrane (right panel). **b** TIRFM imaging of cells expressing CFP-DAT and σ_1_R-YFP reveals localization of the two proteins at or near the plasma membrane (scale bar: 25 μm). **c** Super-resolution STED microscopy of σ_1_R-YFP expressing cells. The inset shows a 200% magnification of the indicated region revealing a σ_1_R-YFP hot-spot at the cellular edge (scale: 5 µm). **d** YFP-DAT cells expressing σ_1_R-CFP shows areas of the endoplasmic reticulum (as indicated by σ_1_R-CFP expression) in close contact with the plasma membrane (as indicated by YFP-DAT expression; scale bar 25 µm). The right panel shows a 500% magnification of the indicated region. Arrows highlight the areas of contact between σ_1_R-CFP and YFP-DAT. **e** FRET efficiency (FRET_eff_) analysis reveals an interaction between σ_1_R-CFP and YFP-DAT. While 1 μM PRE-084 (30 min), 1 μM BD-1063 (30 min), or METH (10 μM, 15 min) alone did not increase the FRET_eff_ compared to control, combined PRE-084 and METH treatment significantly increased the association between σ_1_R-CFP and YFP-DAT as measured by an increased FRET_eff._ 1 μM BD1063 pretreatment before PRE-084/METH blocked the effect of PRE-084. (*n* = 10–19 cells per group; *F*_(5,75)_ = 2.4, *P* = 0.0005, one-way ANOVA; Tukey’s test for multiple comparisons; *P* = 0.1694 comparing PRE-084/METH to BD1063/PRE-084/METH). **f** Nonspecific FRET was determined in cells expressing YFP-DAT/empty CFP-vector or σ_1_R-CFP/YFP-vector (*n* = 5 cells). Additionally, σ_1_R-YFP co-expressed with CFP-transferrin receptor (CFP-TfR) did not result in detectable FRET (*n* = 4 cells). For positive control, the CFP-YFP fusion protein FRET8 was used (*n* = 5 cells). **P < 0.01. Data is represented as mean ± SEM

We next examined whether the σ_1_R associates with the transporter at the plasma membrane, where DAT is functionally relevant. Immunolabeling of fixed dopamine neurons was visualized using both confocal and total internal reflection microscopy (TIRFM) to investigate the cellular distribution of DAT and σ_1_R (Fig. 9a, left panel). Consistent with previous studies, endogenous σ_1_Rs appeared in a clustered pattern throughout the neuron prominently in the neuronal cell bodies and moderately in the neuronal processes^19,53^. Similar to our study, multiple reports have also shown functional DAT expression in the soma cell bodies, and neuronal processes of dopamine neurons^54,55^. Taken together, these reports suggest that the association between the σ_1_R and DAT may potentially occur throughout the entire neuron. The Pearson’s correlation coefficient was measured to quantity the rate of association between the fluorescence signals of σ_1_R and DAT in the neuronal cell bodies, where a Pearson’s value of 1 denotes perfect correlation between the two signals, −1 implies perfect anti-correlation, and 0 implies no correlation^56^. The measured correlation coefficient of 0.534 ± 0.03 (*n* = 24, from four independent experiments) suggests a modest colocalization between the two proteins. Within the same immunolabeled dopamine neurons, we also visualized the expression of the two proteins using TIRFM. Detection of endogenous σ_1_R in the TIRF field support the interpretation that a population of σ_1_Rs may exist at or near the plasma membrane where they could potentially interact with DAT or other membrane proteins (Fig. 9a, right panel).

We next used a variety of additional approaches to further investigate the membrane localization of the σ_1_R. Consistent with TIRFM imaging in immunolabeled dopamine neurons, σ_1_R-YFP/CFP-DAT expressing cells revealed an extensive distribution of σ_1_R-YFP at the cells’ basal surface (Fig. 9b, middle panel), verifying that a population of σ_1_Rs exist at or near the plasma membrane in multiple model systems. Additionally, we utilized super-resolution stimulated emission depletion (STED) imaging in cells expressing σ_1_R-EYFP. Cells were imaged at 30 nm resolution, revealing a clear presence of σ_1_R in “hot-spots” along the cellular edge corresponding with the plasma membrane (Fig. 9c). As shown in Fig. 9d, confocal imaging in YFP-DAT cells expressing σ_1_R-CFP show σ_1_Rs both on the intracellular ER lattice and on several protrusions of the ER proximal to the plasma membrane, as demarcated by the YFP-DAT signal, indicated by arrows in the 500% magnified image. Time-lapse confocal imaging reveals the dynamic nature by which these ER structures associate with the plasma membrane (Supplementary Movie 1). These ER protrusions have been previously described as subsurface cisternae^21^ and represent unique loci at ER–plasma membrane junctions where ER and plasma membrane proteins have been shown to interact^57,58^. Taken together these data support the idea that a population of σ_1_Rs serve as a communication hub between the ER and plasma membrane proteins, whereby it could dynamically modulate DAT function.

### σ_1_R activation and METH increases the association of σ_1_R and DAT

As a complementary approach to examine how both METH and σ_1_R ligands influence the association of σ_1_R with DAT at the plasma membrane, we next performed Förster resonance energy transfer (FRET) acceptor photobleaching at the membrane of living cells. Photobleaching of YFP-DAT at the membrane plane of the cells resulted in a significant increase in σ_1_R-CFP fluorescence above baseline, confirming interactions between σ_1_R and DAT (Fig. 9e). While treatment alone with the σ_1_R agonist PRE-084 (1 µM), antagonist BD1063 (1 µM), or METH (10 µM) did not alter the FRET efficiency compared to untreated cells, combined exposure of PRE-084 and METH significantly increased FRET efficiency between YFP-DAT and σ_1_R-CFP. This suggests that activation of the σ_1_R in the presence of METH promotes σ_1_R association with DAT, a potential cellular mechanism by which σ_1_R activation modulates METH-mediated DAT activity. PRE-084 selectivity for the σ_1_R is supported by blockade of the effect of PRE-084/METH by pretreatment with BD1063. As control experiments, FRET efficiencies were measured in cells expressing either empty YFP or CFP vectors and the respective DAT or σ_1_R FRET partner (Figs. 9f, 1 and 2), as well as cells expressing σ_1_R-YFP and CFP-tagged transferrin receptor, another membrane localized protein (Figs. 9f, 3). No detectable FRET was measured in these experiments, supporting the specificity of σ_1_R-DAT association. Cells expressing YFP-CFP fusion construct (FRET8) that produces high FRET efficiency served as the positive control (Figs. 9f, 4).

### σ_1_R activation decreases METH-stimulated calcium dynamics

**Fig. 10:**
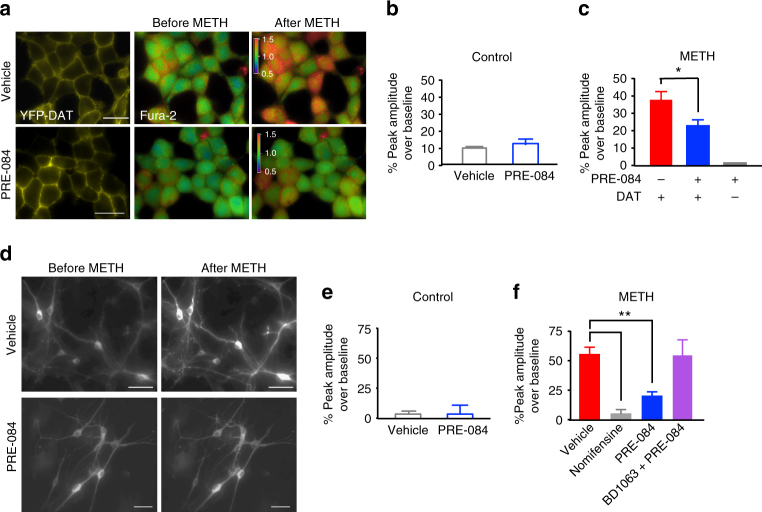
σ_1_R activation significantly decreases METH-induced increases in intracellular calcium. **a** Representative images of YFP-DAT cells (left panel) treated with Fura-2AM before and after treatment with 10 μM METH. Middle and right panel show the ratio 340/380 view. Scale bar is 5 μm. The color bar in the Fura-2 images represent the color assignments for the 340/380 ratio of the cells. **b** PRE-084 did not change calcium levels compared to vehicle treatment. Bar graph shows the average calcium responses after acute vehicle (*n* = 3) or 1 μM PRE-084 treatment (*n* = 3, *n* represents independent experiments including 19–20 cells; *t* = 0.699, *P* = 0.5159, unpaired *t*-test). **c** PRE-084 treatment significantly decreased METH-stimulated intracellular calcium increase compared to vehicle-treated cells. Bar graph shows METH-mediated calcium response over baseline in vehicle treated (*n* = 8) and 1 μM PRE-084 pretreated (30 min) cells (*n* = 8). METH did not increase intracellular calcium in parental cells not expressing DAT (*n* = 5) (*n* represents independent experiments; *F*_(2,18)_ = 3.4, *P = *0.0001, one-way ANOVA, Tukey’s test for multiple comparisons). **d** Grayscale images of vehicle (top panel) or PRE-084 (bottom panel) pretreated neurons expressing the calcium indicator GCaMP6f before and after treatment with 10 μM METH. Scale bar is 20 μm. **e** PRE-084 does not change baseline calcium levels in dopamine neurons. Bar graph shows the average calcium responses after acute vehicle (*n* = 19) or 1 μM PRE-084 treatment (*n* = 11) (*n* represents neurons; *t* = 0.699, *P* = 0.5159, unpaired *t*-test). **f** METH increase intracellular calcium in the dopamine neurons, and this effect is blocked by pretreatment with nomifensine. PRE-084 treatment significantly decreases METH-stimulated intracellular calcium increase compared to vehicle-treated neurons. The effect of PRE-084 was blocked by pretreatment with BD-1063. Bar graph shows METH-mediated calcium response over baseline in vehicle treated (*n* = 53) and 1 μM PRE-084 pretreated (30 min) neurons (*n* = 42). (*n* neurons; *F*_(3, 113)_ = 3.662, *P* = 0.0001, one-way ANOVA, Dunnett’s test for multiple comparisons.) Data is represented as mean ± SEM

Several studies have shown that σ_1_Rs regulate intracellular calcium homeostasis^17,35^. Importantly, DAT-mediated dopamine efflux is calcium-dependent^36^, where reduction of intracellular calcium significantly decreases dopamine efflux; therefore, we investigated whether σ_1_R activation modulates METH-mediated increases in intracellular calcium levels using Fura-2 AM and GCaMP6f. Representative images of Fura-2-treated DAT-expressing cells are shown in Fig. 10a. While neither vehicle nor σ_1_R agonist PRE-084 treatment altered basal calcium homeostasis (Fig. 10b), METH (10 µM) significantly increased intracellular calcium in DAT expressing cells (Fig. 10c). Pretreatment with the σ_1_R agonist PRE-084 resulted in a significant reduction in METH-stimulated increases in intracellular calcium. Consistent with previous studies that METH-mediated increases in calcium are DAT-dependent^40^, METH treatment did not increase intracellular calcium in parental cells not expressing DAT. These experiments were repeated in cultured midbrain neurons using GCaMP6f (Fig. 10d, Supplementary Movie 2). Consistent with findings in DAT cells, METH (10 µM) treatment caused an increase in intracellular calcium, and this response was blocked by pretreatment with the DAT blocker, nomifensine, also suggesting this increase in intracellular calcium is DAT-dependent (Fig. 10f). While PRE-084 alone had no effect on basal calcium homeostasis in midbrain neurons (Fig. 10e), the METH-induced increase in intracellular calcium was prevented by agonist treatment, further supporting the conclusion that σ_1_R activation can attenuate METH stimulation of dopamine neurotransmission. The selectivity of PRE-084 (1 µM) is supported by the blockade of the effect of PRE-084 by the σ_1_R antagonist BD-1063 (1 µM). Collectively, these data provide a novel cellular mechanism for σ_1_R regulation of METH-induced responses, as well as a new molecular target for modulation of METH-stimulated responses. Our proposed working hypothesis is illustrated in Supplementary Fig. 6 and further discussed below.

## Discussion

In this study, we showed that σ_1_R activation attenuates METH-induced cellular and behavioral responses. Extensive evidence suggests σR ligands attenuate the effects of METH^24–29^; however, the potential mechanisms remain unclear. In our studies, we used several approaches including genetic σ_1_R knockout and knockdown, live cell confocal and TIRF microscopy, super-resolution STED microscopy, in vitro and in vivo electrophysiology, behavioral assays, and biochemistry to examine σ_1_R regulation of METH-induced responses. We found that activation of σ_1_Rs decreases METH-stimulated firing of dopamine neurons, dopamine efflux, and intracellular calcium mobilization, all of which are stimulated by METH in a DAT-dependent manner. While our neurochemical and electrophysiological recordings, as well as behavioral experiments provide functional evidence, biochemical, cellular, and molecular approaches provide potential mechanistic information for σ_1_R regulation of DAT-mediated, METH-stimulated responses. Importantly, our data suggest that σ_1_R activation does not affect responses in the absence of METH, i.e. under basal physiological conditions. Only in the presence of METH does σ_1_R activation attenuate METH-induced cellular and behavioral responses. These findings point to the σ_1_R as a potential target for therapeutic intervention in the treatment of METH addiction.

We first genetically and pharmacologically targeted the σ_1_R to examine the role of the σ_1_R on basal and METH-stimulated, DAT-dependent firing activity of cultured dopamine neurons and found that both treatment with the σ_1_R agonist PRE-084 or σ_1_R overexpression decreased METH-stimulated firing of dopamine neurons with no effect on firing activity in the absence of METH. These data are consistent with previous studies reporting no effect of σ_1_R ligands alone^35^, but reported modulatory roles of σ_1_R ligands in potentiating or attenuating stimulated responses. Agonist treatment is known to increase free σ_1_Rs by dissociating it from BiP. Because we did not overexpress BiP when overexpressing the σ_1_R, this hypothetically increases free σ_1_Rs and recapitulates of the agonist-treated state, thereby constituting a necessary component of functional modulation. This occurrence might explain why overexpression and agonist treatment result in the same outcome. When examining knockdown of σ_1_Rs in dopamine neurons by siRNA or utilizing σ_1_R KO mice, neither condition affected basal nor METH-stimulated firing activity of dopamine neurons, suggesting that endogenous σ_1_Rs in the absence of agonist treatment or overexpression is not sufficient for σ_1_R’s modulatory potential of DAT-mediated responses to be realized. This is also consistent with the lack of effect of σ_1_R overexpression or agonist treatment in the absence of METH in which σ_1_R’s modulatory effects may be below the detection limit.

Recordings were repeated in VTA brain slices, and consistent with findings in primary cultures, METH treatment in the midbrain slices significantly increased the firing activity of the dopamine neurons and pretreatment with PRE-084 completely blocked the ability of METH to increase firing activity. Recordings from σ_1_R knockout mice demonstrated PRE-084 pretreatment had no effect on METH-stimulated firing activity suggesting that PRE-084 acts via σ_1_Rs to attenuate this response. Overall, electrophysiological studies support a role for the σ_1_R in modulating METH-stimulated neuronal activity. We and others have shown that METH treatment promotes the inward facing conformation of DAT, leading to an inward DAT-mediated depolarizing current that increases neuronal excitability and intracellular calcium^2,9,40^. As such, our data are consistent with the interpretation that a particular combination of cellular events, such as neuronal depolarization, increased intracellular calcium, and/or conformational changes in DAT, are prerequisite for σ_1_R suppression of METH-stimulated responses. This regulation of METH-induced, but not basal, properties of DA neurons highlights an important therapeutically appealing aspect of σ_1_Rs.

Our time resolved in vitro and in vivo electrochemical analyses suggest that σ_1_R activation does not affect basal DAT-mediated dopamine efflux but attenuates METH-induced dopamine efflux. These data further support the modulatory role of σ_1_R ligands on stimulated responses without effecting basal dopamine neurotransmission in the absence of METH. Furthermore, our findings that PRE-084 pretreatment in DAT cells did not affect METH-inhibition of DAT-mediated uptake or DAT trafficking suggests that σ_1_R regulation of METH-evoked dopamine efflux, and not uptake or trafficking, is a key mechanism for the observed reduction in METH-stimulated responses. Our data are consistent with a recent study by Hong et al. showing that 10 μM PRE-084 (the highest dose used in this study) did not affect dopamine uptake^23^. This study did, however, find that 20 μM PRE-084 increased dopamine uptake in cells overexpressing the σ_1_R that was shown to increase the outward facing confirmation of DAT. The difference in doses as well as σ_1_R expression levels likely contributes to these differing results for dopamine uptake. In addition, considering our finding that PRE-084 decreases METH-stimulated dopamine efflux, the low temporal resolution of radiometric uptake assays may not distinguish the contribution of decreased dopamine efflux on net dopamine remaining in the cell. Utilizing ASP^+^ to examine substrate uptake independent of DAT-mediated efflux, we showed that PRE-084 does not affect substrate uptake alone or in the presence of METH. Overall, our findings suggest that at the doses of PRE-084 used in vitro in the current study, the σ_1_R agonist does not affect basal DAT function but inhibits DAT-mediated, METH-stimulated efflux.

DAT-dependent, METH-stimulated dopamine efflux and firing activity promote a collection of behavioral responses to METH. Given our findings that PRE-084 decreases these effects, we examined whether σ_1_R agonism attenuates METH-induced acute hyperactivity, as well as reinforcing behaviors and found that treatment with the σ_1_R agonist PRE-084 inhibits METH-stimulation of locomotor activity, decreases the acquisition of METH-induced CPP, and attenuates the effect of METH on decreasing brain reward thresholds. Although these findings are consistent with studies that have used σ_1_R agonists with high selectivity for the σ_1_R over σ_2_R (PRE-084 has three hundred times higher affinity for the σ_1_R over σ_2_R)^28^, these findings differ from studies using other σR ligands. This may be due to differences in the doses or type of σ_1_R ligand used, including compounds nonselective for σ_1_R over σ_2_R or doses of σR ligands that may have potential off-target effects, limiting the comparison across these studies. Another important source of variation across these studies may be the psychostimulant being investigated (e.g. cocaine vs. METH). Mechanistic differences between cocaine and METH make it very likely that the mechanism/s for σ_1_R regulation of METH-induced DAT responses differs from that of cocaine. This is especially important to consider because we found that σ_1_R activation decreases METH-stimulated firing activity and dopamine efflux, mechanisms that are blocked by cocaine.

One unexpected but intriguing finding was that treatment with 30 mg/kg, but not 10 mg/kg, of the σ_1_R antagonist BD1063 suppressed basal locomotion during the first 15 min following BD1063 pretreatment. Comparison of locomotor activity over a full hour following BD1063 (30 mg/kg BD1063 + saline group) was not different from control mice (saline + saline group), suggesting an acute sedative effect of BD1063 similar to properties of other σ_1_R antagonist reported previously^42,43^. Not surprisingly, 30 mg/kg of BD1063 in combination with METH elicited a similar attenuation of METH-induced hyperlocomotion as did the highest dose of PRE-084 tested (8 mg/kg). This dose of PRE-084 had no effect on basal locomotor activity, suggesting that unlike BD1063, PRE-084 does not have baseline sedative effects. While BD1063 blocked PRE-084’s effects in other assays used in this study, BD1063’s potential sedative effects at 30 mg/kg make it not surprising that this dose was not able to block the effect of 8 mg/kg PRE-084 and suggest more complex mechanisms of action. Although 10 mg/kg of BD1063 was not able to completely block the effect of 8 mg/kg PRE-084 on METH-induced locomotor activity, partial blockade occurred towards the end of the 60-min period. One possible explanation is that 10 mg/kg of BD1063 fails to reach the brain in levels sufficient to fully antagonize σ_1_Rs, supported by the lack of effect of 10 mg/kg but noticeable effect of 30 mg/kg BD1063. Only in vivo pharmacokinetic studies could properly address this possibility.

Beyond investigating the functional effects of σ_1_R activation on METH-mediated activity, we aimed to identify cellular mechanism/s by which σ_1_R activation inhibits METH dysregulation of dopaminergic neurotransmission. Consistent with a recent report^23^, we found that the σ_1_R associates with DAT and further that this σ_1_R/DAT complex exists at or near the plasma membrane in areas of the ER proximal to the plasma membrane called subsurface cisternae^19,20^. These ER structures are important in regulating various cellular processes including membrane protein insertion, protein trafficking, intracellular calcium homeostasis, and facilitating points of contact between ER proteins and plasma membrane proteins^58–61^. Previous studies have detected σ_1_R within the plasma membrane fraction of cells via biochemistry^17,35,62^ or showed the localization near the plasma membrane via electron microscopy^19,20^. Additionally, Kourrich et al. showed that the σ_1_R can fully integrate into the plasma membrane, providing a potential mechanism by which σ_1_Rs regulate transmembrane proteins. Therefore, σ_1_Rs may represent an important conduit of intracellular communication between the ER and plasma membrane. Our FRET data support this interpretation, where only in the combined presence of the σ_1_R agonist PRE-084 and METH was the DAT/σ_1_R complex increased. This idea is consistent with reports that σ_1_R activation alone does not elicit cellular or behavioral responses but stimulated or pathological conditions provoke σ_1_R modulation of activity, in this case its interaction with DAT. Thus, activation of σ_1_Rs by PRE-084 in the presence of METH may promote σ_1_R association with DAT, thereby influencing DAT activity. This could occur via modification of the conformational state of DAT, altering post-translational modifications, and/or altering DAT interactions with other proteins, which all could decrease METH-induced DA efflux^63^. Currently, it is unknown whether the proteins directly interact or are associated via a larger protein complex. Furthermore, the role of basal interactions between the σ_1_R and DAT has yet to be determined. One intriguing hypothesis, however, is that endogenous σ_1_R associations with DAT under basal conditions is a physical-but-not-functional interaction unless multiple events requiring both agonism and concomitant METH-stimulated responses (i.e. neuronal depolarization, increased intracellular calcium, inward facing conformation of DAT) are present.

While σ_1_R associations with DAT may serve as a potential mechanism by which the σ_1_R modulates DAT activity, the σ_1_R may also modulate DAT activity via indirect mechanisms. Reports suggest that σ_1_Rs can buffer intracellular calcium^17,35^, and METH-stimulated dopamine efflux and modulation of firing activity of dopamine neurons are calcium dependent^36^. Therefore, σ_1_R inhibition of METH-stimulated calcium mobilization is another potential mechanism underlying the attenuation METH-mediated dopamine efflux, excitability of dopamine neurons, and behavioral responses reported here. Alternatively, it is possible that ligand activation of σ_1_Rs may in turn influence activity of K^+^ channels to indirectly decrease DAT activity through the modulation of membrane potential. This might explain the decreased excitability of dopamine neurons after METH treatment when σ_1_Rs are overexpressed or after σ_1_R agonist exposure. In addition to interactions with K^+^ channels, σ_1_Rs have been shown to interact with D1 and D2 receptors^30,32^. While interactions with these receptors may also play a role in the effects of METH, these effects would be subsequent to the effects of σ_1_Rs on METH-mediated DAT activity. Because of the wide scope of cellular functions regulated by σ_1_Rs, it may also indirectly modulate DAT activity through other signaling cascades, such as altering kinase activity and membrane lipid redistribution, both of which are characterized actions of σ_1_Rs^17,18,35^ and are known to regulate DAT activity^36,64^. Overall, given the different independent mechanisms of actions of METH on DAT-activity, as well as the diversity of σ_1_R functions, defining the direct molecular mechanism of σ_1_R activation on METH-mediated DAT activity requires further investigation, especially considering multiple molecular mechanisms are likely acting simultaneously. The proposed mechanisms are illustrated in Supplementary Fig. 6.

In conclusion, our findings show that σ_1_R activation attenuates DAT-mediated, METH-stimulated increases in firing activity of dopamine neurons, dopamine efflux, and behavioral responses without affecting dopamine uptake or DAT trafficking. Notably, we found that the σ_1_R agonist PRE-084 has neither aversive nor rewarding properties and does not influence basal dopamine transmission. Findings that PRE-084 treatment in the presence of METH increases the interaction between σ_1_R and DAT, as well as PRE-084’s ability to reduce METH-stimulated calcium increases, provide potential cellular mechanisms for the actions of σ_1_R to attenuate METH-induced enhancement of dopamine neurotransmission. Taken together, these studies encourage further examination of the σ_1_R as a target for development of novel therapeutics to treat METH addiction.

## Methods

### Materials

EYFP tagged σ_1_R (σ_1_R-EYFP), GFP tagged σ_1_R siRNA (GFP-σ_1_R siRNA, under the U6-C6-CMV ubiquitous promoter), and inactive siRNA plasmids were generous gifts from Dr. Tsung Su (NIDA NIH). Cerulean fluorescent protein tagged σ_1_R (σ_1_R-CFP) was generated by substituting EYFP with CFP in the σ_1_R-YFP plasmid. The CFP-DAT plasmid was a generous gift from Dr. Alexandar Sorkin (University of Pittsburg). FRET8 was a generous gift from Dave Piston (Vanderbilt University). CFP-Transferrin Receptor was obtained from AddGene, and AAV1.syn.GCaMP6f.WPRE.SV40 was obtained from the University of Pennsylvania Penn Vector Core. Primary antibodies were obtained as listed in Supplementary Table 1. Secondary antibodies were obtained from Life Technologies, unless specified. PRE-084, BD-1063, nomifensine, sulpiride, SCH23390, and METH were purchased from Sigma Aldrich. All other chemicals and materials were purchased as indicated.

### Animals

Wild-type (WT) male C57BL/6J mice were obtained from the University of Florida Animal Care Services for behavioral experiments. σ_1_R knockout mice were obtained from the Mutant Mouse Resource & Research Centers at the University of California, Davis^65^. Mixed background σ_1_R knock-out mice (129S5/SvEvBrd and C57BL6/J; UC-Davis MMRRC stock #011750-UCD) were used for all experiments with the exception of the slice electrophysiology experiments which utilized congenic σ_1_R knock-out mice backcrossed to C57BL/6J for 10 generations (KO; UC-Davis MMRRC stock #036775/UCD). Female and male pups were mixed to generate primary culture. Male Wistar rats were obtained from Charles Rivers Laboratories. Animals were housed in the animal care facilities and maintained as approved by Institutional Animal Care and Use Committee of the University of Florida and followed by guidelines established by National Institutes of Health. Mice were housed at 3–5 per cage, and rats were housed at two per cage. Food and water were available ad libitum in the home cage. The room was maintained under 12-h light/dark cycle.

### Primary neuronal culture

Acutely dissociated mouse midbrains from 0 to 2-day-old male and female pups were isolated and incubated in dissociation medium (in mM): 116 NaCl; 5.4 KCl; 26 NaHCO_3_; 25 d-glucose; 2 NaH_2_PO_4_; 1 MgSO_4_; 1.3 cysteine; 0.5 EDTA; 0.5 kynurenate containing 20 units/ml papain at 34–36 °C under continuous oxygenation for 2 h. Tissue was triturated with a fire-polished Pasteur pipette in glial medium (in %): 50 minimum essential medium; 38.5 heat-inactivated fetal bovine serum (FBS); 7.7 penicillin/streptomycin; 2.9 d-glucose (45%) and 0.9 glutamine (200 mM). Dissociated cells were pelleted by centrifugation at 500×*g* for 5 min and resuspended in glial medium. Cells were plated on 12 mm coverslips coated with 100 μg/ml poly-l-lysine and 5 μg/ml laminin. Two hours after plating, the medium was changed to neuronal medium (in %): two minimum essential medium, 75 hams-F12 medium, 19 heat-inactivated horse serum, two heat-inactivated FBS, 1.56 d-glucose (45%), 0.04 insulin (0.025 g/ml), and 0.4 apotransferrin (50 mg/ml). Neuronal medium was conditioned overnight on cultured glia. Conditioned neuronal medium was supplemented with 1 ng/ml glial cell line-derived neurotrophic factor and 500 μM kynurenate.

### Cell culture

Chinese Hamster Ovary (CHO) and Human Embryonic Kidney (HEK) cell lines stably expressing YFP-DAT or FLAG-DAT were generous gifts from Dr. Jonathan Javitch (Columbia University). YFP-DAT CHO cells were used for microscopy experiments because they are flat and have minimal basal fluorescence^66^. YFP-DAT and FLAG-DAT HEK cells have been widely used in the field to study DAT biology. CHO cells were maintained in Ham’s F12, and HEK cells were maintained in DMEM. Cell lines were checked for mycoplasma using a standard mycoplasma detection kit. Media was supplemented with 10% FBS and 5% l-glutamine. Cells were maintained at 37 °C and 5% CO_2_ and typically used for experiments after reaching 60–80% confluency. To induce expression of proteins not stably expressed, cells were transfected using a standard lipofectamine 2000 protocol, and transfected cells were used in experiments 12–36 h after transfection.

### Electrophysiological recordings

Spontaneous firing of primary culture dopamine neurons 8–12 days in vitro was examined via whole cell current clamp recordings. Dopamine neurons were identified morphologically, by their large cell bodies with broad first-order processes, pharmacologically, and electrophysiologically. Neurons were continuously perfused with aCSF containing (in mM): 126 NaCl, 2.5 KCl, 2 CaCl_2_, 26 NaHCO_3_, 1.25 NaH_2_PO_4_, 2 MgSO_4_, and 10 dextrose, equilibrated with 95% O_2_−5% CO_2_, pH 7.4. Experiments were performed at 37 °C. Patch electrodes were fabricated from borosilicate glass (1.5 mm outer diameter; World Precision Instruments) with the P-2000 puller (Sutter Instruments). The tip resistance was in the range of 3–5 Ω. The electrodes were filled with pipette solution containing (in mM) 120 potassium-gluconate, 20 KCl, 2 MgCl_2_, 10 HEPES, 0.1 EGTA, 2 ATP, and 0.25 GTP, pH 7.35. During recordings, drugs were applied at concentrations previously been shown to be effective without cell toxicity^9,10,40^. For neurons expressing σ_1_R-YFP, GFP-σ_1_R siRNA, and inactive siRNA, plasmids were transfected via Calcium Phosphate Transfection Kit (Life Technologies) per manufacturer instructions. Transfection did not affect the resting membrane potential (−51.0 + 1.6 mV transfected neurons vs. −50.3 + 1.4 mV non-transfected neurons); basal frequency of spontaneous firing activity (1.02 + 0.07 transfected neurons vs. 1.07 + 0.08 non-transfected neurons); and neuronal morphology. siRNA knockdown of σ_1_R in HEK cells was validated by Western blot analysis. The Western blot methods are described below. Rabbit anti-σ_1_R was used to detect σ_1_R levels, mouse anti-GFP for expression of siRNA, and mouse anti-β-actin as a loading control. Images have been cropped for presentation. Full size images are presented in Supplementary Fig. 7.

For ex vivo VTA recordings, adult postnatal day 35–40 male C57BL/6J WT and congenic σ_1_ R knock-out mice (KO; UC-Davis MMRRC stock #036775/UCD) were used. Animals were housed under standard conditions at 22−24 °C, 50–60% humidity, and a 12 h light/dark cycle. Mice were deeply anesthetized with 4% isoflurane and the brain was removed from the cranium. The tissue was glued onto the cutting stage and submersed in ice-cold, oxygenated aCSF containing (in mM) 126 NaCl, 2.5 KCl, 2 CaCl_2_, 26 NaHCO_3_, 1.25 NaH2PO_4_, 2 MgSO_4_, and 10 dextrose, equilibrated with 95% O_2_–5% CO_2_, and pH adjusted to 7.4. Coronal midbrain slices (250 μm) containing the VTA were cut using a vibratome 1000 (Vibratome 1000 plus; Ted Pella Inc.) and maintained in an interface chamber filled with aCSF. Slices were transferred to a recording chamber maintained at room temperature (22–25 °C). To record spontaneous firing activity of VTA dopamine neurons, the chamber was superfused with aCSF solution as described above at a speed of 2 ml/min. VTA neurons were visualized on an upright microscope (Nikon ECLIPSE FN1) using infra-red differential interference contrast Dodt tube optics. Unless indicated, all experiments were performed in the presence of the following antagonists in the aCSF: 5uM R(+)-SCH-23390 (D_1_ receptor), 5 μM Sulpiride (D_2_ receptor), 10 μM SR-95531 (GABA_A_ receptor), 100 nM CGP 35348 (GABA_B_-receptor), 20 μM MK-801(NMDA glutamate receptor), and 10 μM CNQX (AMPA/kainate glutamate receptor). The electrode resistance for all experiments was 3–5  MΩ. The internal pipette solution was the same as described for primary culture neuronal recordings.

### Amperometry recordings

DAT-mediated dopamine efflux was monitored in YFP-DAT HEK cells. The amperometric carbon fiber electrode (ProCFE, Dagan Corp.) was connected to a second amplifier (Axopatch 200B, Molecular Devices, Sunnyvale, CA) and attached to the plasma membrane of the cell and held at 700 mV. The diameter of the carbon fiber electrode was 5 µm. Two millimolars of dopamine was dialyzed into the cell via the patch electrode, and an oxidative (amperometric) current was generated at membrane potentials between +80 and +100 mV. The resulting amperometric current is reported directly without considering the effective volume. METH-induced, DAT-mediated dopamine efflux was determined by subtracting the baseline current from the amperometric current recorded after bath application of 10 μM METH. The steady-state current was calculated as the average current during the final 100 ms of each potential tested. For experiments with the σ_1_R agonist, cells were pretreated with 1 μM PRE-084 in external solution for 30 min prior to amperometry recordings. For experiments with combined σ_1_R agonist and antagonist, cells were pretreated with 1 μM BD-1063 for 10 min followed by additional treatment with 1 μM PRE-084 for 30 min prior to recordings.

### [^3^H]DA uptake assay

FLAG-DAT HEK cells were plated at 100,000 cells/well and grown for 72 h. Cells were washed twice with Krebs–Ringer solution (KRH) that contained (in mM): 125 NaCl, 12 Na_2_HPO_4_, 4.8 KCl, 1.2 MgSO_4_, 1.3 CaCl_2_, 5.6 d-glucose, 25 HEPES, and 0.05 ascorbic acid (pH 7.4). Cells were pre-treated with 0.01–10 μM PRE-084 or 0.001–1 μM BD-1063 for 30 min. For METH treatments, cells were incubated with 10 µM METH for 15 min followed by three KRH washes. Uptake of 10 nM [^3^H]DA plus 3 µM cold DA was carried out for 10 min at RT. The concentration of dopamine was determined in preliminary dose-responses experiments (Supplemental Fig. 1A) where the concentration of cold dopamine at half maximum was further used. Uptake was terminated with three washes in ice-cold PBS, and cells were lysed with 10% SDS. Nonspecific uptake was determined in cells pretreated with 10 µM nomifensine for 10 min. Radioactivity was measured in a liquid scintillation counter (LS 5801, Beckman Coulter).

### ASP^+^ uptake assay

Experiments were performed in YFP-DAT HEK cells as previously described via a Nikon A1 laser-scanning confocal microscope (Nikon Corporation) at 37 °C^67^. Cells were imaged in a standard external solution containing (in mM): 130 NaCl, 10 HEPES, 34 dextrose, 1.5 CaCl_2_, 0.5 MgSO_4_, and 1.3 KH_2_PO_4_ at pH 7.35 and osmolarity of 270 mOsm. Images of YFP-DAT were taken at the beginning of each experiment. 4-Di-1-ASP (4-(4-(dimethylamino)styryl)-N-methylpyridinium Iodide) (ASP^+^) (Life Technologies) was dissolved in external solution. To measure ASP^+^ uptake, florescence was measured at an excitation of 488 nm and emissions of 590/50 nm. Baseline images were acquired at 1 Hz for 10 s, followed by the addition of ASP^+^ solution to give a final concentration of 2 μM. Images were acquired for 300 s. For drug treatments, cells were treated with 1 μM PRE-084 for 30 min and/or 10 μM METH for 15 min at 37 °C. For analysis, regions of interest (ROIs) were detected on YFP-DAT images to encompass to entire internal region of the cell. Background fluorescence was subtracted from each frame. Uptake of ASP^+^ was determined from the average fluorescent units normalized to the average fluorescence of the first 10 s of baseline imaging for each cell.

### In vivo high-speed chronoamperometry

Experiments were conducted using the FAST-12 system (Quanteon) as previously described with minor modifications^68,69^. Carbon fiber recording electrodes were coated with 5% Nafion (Aldrich Chemical Co.) to provide a 1000-fold selectivity for dopamine over its metabolite dihydroxyphenylacetic acid (DOPAC). Under these conditions, microelectrodes displayed linear amperometric responses to 0.5–10 µM DA during in vitro calibration in 100 mM phosphate-buffered saline (pH 7.4).

Carbon fiber microelectrodes were attached to glass multi-barrelled micropipettes (FHC) such that the distance between the microelectrode and micropipette tips was 200 mm. Barrels of the micropipette were filled with either METH (800 µM; (+)-METH), PRE-084 (100 µM) or vehicle (aCSF). Adult male C57BL/6J mice were anesthetized by i.p. injection (2 ml/kg body weight) of a mixture of urethane (250 mg/kg) and a-chloralose (25 mg/kg), followed by tracheal intubation to facilitate breathing, and placed into a stereotaxic frame (David Kopf Instruments). The electrode/micropipette assembly was lowered into the dorsal striatum at the following coordinates (in mm) from bregma: A/P + 1.1; M/L+/− 1.4; D/V −2.25 mm to −2.70. Body temperature was maintained at 36–37 °C and blood oxygen levels monitored (MouseOximeter, StarrLifeSciences) and maintained above 90%.

Drugs were pressure-ejected into dorsal striatum using a Picospritzer II (General Valve Corporation) in an ejection volume of 125 nl (METH) or 100 nl (PRE-084) (5–25 psi for 0.25–3 s) according to the following sequence: First, METH (100 pmol) was pressure-ejected, producing robust DA release ranging from ~0.5 to 2.0 µM peak signal amplitude. A period of 45 min was permitted to elapse to allow releasable pools of DA to be restored^68^. Then either PRE-084 (10 pmol) or BD1063 (10 pmol) or vehicle was pressure-ejected. Note that 2 min prior to either PRE-084 or aCSF ejection, aCSF was also given. This is to facilitate comparison of present data with future studies investigating a variety of drug pre-treatments prior to PRE-084. Fifteen minutes later, the same pmol amount of METH was again pressure-ejected, and then again 45 min later.

After ejection of drugs into brain, there is an estimated 10–200-fold dilution caused by diffusion through the extracellular matrix to reach a concentration of 4–80 µM (for METH) or 1–10 µM (for PRE-084) at the recording electrode^70^. The concentration range for METH is consistent with those measured in brain following behaviorally relevant doses^71^. To record the efflux and clearance of dopamine at the active electrode, oxidation potentials consisting of 100 ms pulses of 550 mV, each separated by a 900 ms interval during which the resting potential was maintained at 0 mV, were applied with respect to a Ag/AgCl reference electrode implanted into the contralateral superficial cortex. Oxidation and reduction currents were digitally averaged during the last 80 ms of each 100 ms voltage pulse. For each recording session, dopamine was identified by its reduction/oxidation current ratio: 0.50–0.90.

At the conclusion of each experiment, an electrolytic lesion marked the placement of the recording electrode tip. Mice were decapitated while still anesthetized, and their brains removed, frozen, and stored at −80 °C until sectioned (20 µm) for histological verification of electrode location within the striatum. In addition to reduction/oxidation ratios, five signal parameters were analyzed: (i) maximal signal amplitude of amphetamine-evoked DA release (in mM); (ii) time to reach maximal signal amplitude (rise time, in s); (iii) DA efflux rate (in nM/s), which is the change in DA oxidation current evoked by amphetamine application as a function of time; (iv) clearance time, the time for released DA to be cleared by 80% of maximal amplitude (*T*_80_, in s); and (v) DA clearance rate (in nM/s), defined as the slope of the linear portion of the current decay curve, i.e., from 20 to 60% of maximal signal amplitude.

### Locomotor activity

Drug naïve male C57BL/6 mice 7–8 weeks of age were used for these studies. All experiments were performed during the light cycle. Animals were handled by the experimenter during the light phase for 5 consecutive days followed by habituation to the behavior room and behavioral equipment for 3 consecutive days. Behavioral measures were measured via the Versamax Animal Activity Monitor chambers (AccuScan Instruments) as previously described^72^. On test days, rodents were habituated to the locomotor chamber for 30 min prior to treatment. Animals were given i.p. injection of 1, 2, 4, or 8 mg/kg PRE-084, 10 or 30 mg/kg BD1063, or equivalent volume of saline and locomotor activity was measured for 15 min. Rodents then received a second injection of 2 mg/kg METH or saline and placed in activity monitor chambers for 60 min. Locomotor activity counts recorded for 1 h in 60-s bins. To test the effect of the σ_1_R antagonist BD1063 on PRE-084 reduction of METH-stimulated firing activity, after the 30-min habituation mice received saline or 10 or 30 mg/kg of BD1063 and were returned to the locomotor chamber for 15 min. Rodents then received saline or 8 mg/kg of PRE-084 followed by another 15 min in the locomotor chamber. Rodents lastly received saline or 2 mg/kg of METH, and locomotor activity was recorded for 60 min.

### Conditioned place preference

Drug naïve male C57BL/6 mice 7–8 weeks of age were used for these studies and were handled and habituated as described above. All experiments were performed during the light cycle. Three compartment conditioned place preference (CPP) chambers were used as previously described (Med Associates Inc)^73^. Briefly, one side of the choice chamber had black walls and a rod floor and the opposite side had white walls and a mesh floor. The small center chamber had grey walls and a smooth floor. After habituation, animals underwent the CPP Pre-Test (Day 1) in which animals were given free access to the entire chamber, and time spent on either side (black vs. white) was measured. Preference is measured as time spent on either side. The less preferred side was designated as the drug-paired side, and the more preferred side was designated the saline-paired side. Animals that exhibit inherent bias for one side (spent over 60% of their time on one side of the chamber during the pre-test) were not used in any CPP experiments. Conditioning occurred from days 2 to 9. For dose–response studies, on even days animals received intraperitoneal (i.p.) injections of saline and were placed on the saline-paired side for 20 min. On odd days, animals received either saline, PRE-084 (1, 2, or 4 mg/kg), or METH (1, 2, or 4 mg/kg) by i.p. injection and placed on the drug-paired side for 20 min. To study the effect of PRE-084 on METH-induced CPP, animals received two i.p. injections of saline 15 min apart on even days followed by placement on the saline-paired side. On odd days, animals received i.p. injection of either saline or PRE-084 (1, 2, or 4 mg/kg) followed by injection of saline or 2 mg/kg METH 15 min later. Animals were then placed on the drug-paired side for 20 min. After conditioning, the CPP score was determined on the Preference Test on day 10. Animals were given free access to the entire chamber and time spent on either side was measured. The CPP score was calculated as the time spent on the drug-paired side minus time spent on the saline-paired side.

### Intracranial self-stimulation

Drug naïve Wistar rats at 7 weeks of age were used for all experiments. All experiments were performed during the light cycle. Surgical procedures were conducted as described previously^74–76^. The rats were anesthetized with isoflurane and prepared with electrodes (Plastic One) in the medial forebrain bundle (anterior–posterior −0.5 mm; medial–lateral ± 1.7 mm; dorsal–ventral −8.3 mm from dura). The electrodes were permanently secured to the skull using dental cement. Rats were trained on a modified discrete-trial ICSS procedure as described previously. The operant conditioning chambers were housed in sound-attenuating chambers (Med Associates). The operant conditioning chambers had a 5 cm wide metal response wheel centered on a sidewall and a photobeam detector recorded every 90° of rotation. Brain stimulation was delivered by constant current stimulators (Model 1200C, Stimtek). The rats were trained on a discrete-trial current-threshold procedure. Each test session provided a brain reward threshold and response latency. The brain reward threshold was defined as the midpoint between stimulation intensities that supported responding and current intensities that failed to support responding. The response latency was defined as the time interval between the beginning of the non-contingent stimulus and a positive response. METH was administered subcutaneously (sc) and PRE-084 was administered intraperitoneally (i.p.). METH was given sc to replicate the methodology of a previous study^50^. To determine the acute effect of METH on ICSS, the rats received METH (0, 0.03, 0.1, 0.3, or 0.6 mg/kg) 10 min before ICSS testing when the ICSS thresholds were stable (<10% variation over 5-day period). The drugs were administered according to a Latin-square design. To determine the effect of PRE-084 on METH ICSS, METH (0.3 mg/kg, sc) was administered 10 min before ICSS testing and PRE-084 (1, 3, 9, or 18 mg/kg, ip) was administered 15 min before the administration of METH or saline. PRE-084 was administered according to a Latin-square design. One dose of PRE-084 (18 mg/kg) was added after the Latin-square to complete the dose–response curve. The ICSS parameters (brain reward thresholds and response latencies) were expressed as a percentage of the pre-test day values (day before the drug injections).

### Cell surface biotinylation

FLAG-DAT HEK cells were pretreated with 100 nM or 1 μM of PRE-084 for 30 min and/or 10 μM METH for 15 min at 37 °C in standard external solution. Following treatment, cells were washed three times in PBS. All remaining steps were performed on ice to prevent further trafficking. Surface DAT levels were isolated by treatment with 1.0 mg/ml sulfo-NHS-SS-biotin (Thermo Scientific) for 20 min. Excess biotin was quenched by incubating cells with three washes in 100 mM glycine in PBS. Cells were lysed in RIPA buffer containing protease inhibitors, and 500 μg protein was reacted with neutravidin beads (Thermo Scientific) rotating overnight. Beads were then pelleted and washed three times in RIPA buffer. Protein was eluted in Laemmli Sample Buffer (BioRad) and incubated for 15 min at 65 °C. Samples, excluding beads, were resolved on 10% SDS-PAGE, and proteins were transferred to 0.2 µm PVDF membranes. Membranes were blocked for 1 h at RT in 5% milk followed by overnight incubation with Rat anti-DAT overnight at 4 °C. Membranes were then incubated with HRP-conjugated goat anti-rat secondary antibody for 1 h at RT. Membranes were scanned after incubation with West Pico Chemiluminescent Substrate (Thermo Scientific). Data were analyzed via Bio-Rad GS-800 Densitometer. Images have been cropped for presentation. Full size images are presented in Supplementary Fig. 8.

### Total internal reflection fluorescence microscopy (TIRFM)

YFP-DAT CHO cells were plated on glass-bottom dishes. All TIRFM imaging was completed on a Nikon Ti Eclipse inverted microscope equipped with a multi-line solid-state laser source (470, 514, 561 nm). Lasers were guided through a 60 × 1.4 NA objective. Images were detected digitally using a CCD camera in standard external solution. Image exposure time was coupled with stimulation duration at 100 ms. For PRE-084 treatment, cells were treated with 1 µM PRE-084 for 2 h at 37 °C prior to imaging. For quantification of fluorescence intensity at the cell surface, experimenter-defined ROI were created for each cell, excluding regions of cell overlap and peripheral edges of cells. Background fluorescence was subtracted from all frames. Mean intensity over time for each cell’s ROI was recorded continuously before and after the application of 10 µM METH. All quantification is expressed normalized to the intensity 1 min prior to drug application. Single count elementary sequential smoothing was applied for image presentation only and not analysis. Images are presented in grayscale.

### STED microscopy

Parental HEK cells were grown on 12 mm glass coverslips and transiently transfected with σ_1_R-YFP before being fixed and mounted in Fluoromount-G (Southern Biotech). STED microscopy was carried out using a commercial STED system (Abbrior Instruments) and accompanying software. YFP excitation was achieved with a 485 nm laser; STED was accomplished using a 595 nm laser operating at 8.8 mW at the plane of the sample. YFP emission was captured using a 525/50 nm collection filter. For imaging, the pixel size was 20 nm with 10 µs dwell time and a 0.93 AU pinhole. Images were colorized and converted to 8-bit TIFF files using FIJI^77^.

### Immunofluorescence staining and colocalization analysis

Primary cultured midbrain neurons were washed three times with ice-cold DPBS before being fixed with 3.7% paraformaldehyde prepared from 37% stock (Electron Microscopy Sciences) for 20 min at RT. Samples were blocked and permeabilized in PBS with 10% normal goat serum (Lampire Biological Laboratories) and 0.5% Triton X-100 (Fisher Scientific) for 30 min at RT. Neurons were incubated with rat anti-DAT antibody (Millipore, MAB369, 1:500) and rabbit anti-σ_1_R (Life Technologies, 42–3300, 1:50) in PBS with 5% goat serum and 0.1% Triton X-100 at 4 °C overnight. Neurons were washed three times for 20 min in PBS before incubation with Alexa Fluor^®^ 488 Goat Anti-Rat IgG (H+L) and Alexa Fluor^®^ 568 Goat Anti-Rabbit IgG (H+L) (both Life Technologies, 1:500) in PBS with 5% goat serum and 0.1% Triton X-100. Neurons were washed in PBS overnight before being mounted with Fluoromount-G (Southern Biotechnologies). Confocal images were collected using a Nikon A1 laser-scanning confocal microscope (Nikon Corporation) using a 1.4 NA 60X oil-immersion objective. Images were acquired using a z-series (0.25 µm step size) and 488-nm 561-nm excitation in non-overlapping series with emission captured at 525 and 595 nm, respectively. For the calculation of the Pearson’s correlation coefficient, a single plane was selected from the z-series taken that was proximal to the basal membrane of the cultured DA neurons and fluorescence intensities were measured in the cell body of this single plane. Calculation of the Pearson’s value for individual neurons was carried out in Nikon Elements imaging software (Nikon Corporation), and these were averaged to provide the presented value. TIRF images were captured immediately following confocal imaging on the same neuron. Images were captured using the 488 and 561 nm laser lines in non-overlapping series as above. All images were processed using the Nikon Elements imaging software.

### Co-immunoprecipitation (co-IP)

HEK293 were grown in 12-well culture plates and transfected using GeneCellin (Bulldog Bio) and 1.4 µg plasmid/well (pcDNA3-mDAT, pBABE-σ_1_R, and pcDNA3-σ_1_R-EYFP). Transformed cells were selected with 800 µg/ml Geneticin (G418) (Sigma Aldrich) 36–48 h later. Expression of stable cells was confirmed by Western blot. For co-IP experiments, stable cell lines were plated on 60 mm plates and transiently transfected 48 h prior and maintained under selection with 400 µg/ml Geneticin (G418). Co-IP experiments were carried out as outlined by the manufacturer (Co-Immunoprecipitation Kit; Thermo Scientific). Immunoprecipitation of equal amounts of mDAT, σ_1_R, and σ_1_R-EYFP expressing lysates was conducted for 14–18 h at 4 °C using AminoLink Plus Coupling Resin (ThermoFisher) in serum containing rabbit polyclonal antibodies against DAT (Poly16) raised in (kind gift from Dr. Roxanne Vaughan^78^, rabbit serum containing Sigma Receptor (σ_1_R) antibodies (kind gift from Dr. Arnold Rhouho) or Living Colors^®^ A. V. mouse monoclonal antibody against EYFP (JL-8) (Clontech) to a final concentration of 2 µg. IP and total lysates were subjected to electrophoresis on 10% NEXT GEL^®^ polyacrylamide gels (VWR Internationals) and transferred to 0.2 µm PVDF membranes. DAT, σ_1_R, and EYFP were detected using mouse monoclonal antibody 16 (mAB), mouse monoclonal σ_1_R (B-5) antibody, and rabbit polyclonal Living Colors^®^ A. V. anti-EYFP antibody (Clontech). Bound 1° antibodies were detected with polyclonal goat anti-mouse antibodies (A3562, Sigma Aldrich) (DAT, σ_1_R) or goat-anti-rabbit polyclonal antibodies (EYFP) linked to alkaline phosphatase (A6387, Sigma Aldrich) or horseradish peroxidase (Millipore). Rabbit polyclonal *Drosophila* SERT antibodies (13-A) were used as the unrelated antibody control (Alpha Diagnostics Inc.). The mock control was parental HEK-293 cells that have endogenous σ_1_R but do not express DAT. Images have been cropped for presentation. Full size images are presented in Supplementary Figs. 9 and 10.

### Fluorescence resonance energy transfer (FRET)

HEK cells expressing YFP-DAT and/or σ_1_R-CFP were grown on glass coverslips. FRET was measured via Nikon A1 confocal microscope using a 60X oil-immersion objective. For all experiments, images were taken at 37 °C in standard external solution as described above. Control experiments were performed in HEK cells expressing empty YFP vector with σ_1_R-CFP or empty CFP vector with YFP-DAT. An additional negative control was performed using σ_1_R-YFP and CFP-tagged transferrin receptor. As a positive control, the CFP-YFP fusion protein FRET8 was used. Filter wheels were configured as follows: CFP (donor; excitation 457 nm; emission 464–499 nm), and YFP (acceptor; excitation, 514 nm, emission, 552–617 nm). Fluorescence intensity was measured as arbitrary fluorescence units (AFU). Background fluorescence was subtracted from all images. FRET values were calculated from the mean fluorescence intensities for each experimenter-defined region of interest (ROI) at the plasma membrane. FRET efficiency (FRET_eff_) values for individual ROIs were calculated according to the following equation:

$${\rm FRET_{eff} = \left( {\left( {Donor_{Post}-BkGrd_{Post}} \right) - \left( {Donor_{Pre} - BkGrd_{Pre}} \right)} \right)} \\ / {\rm Donor_{Post}-BkGrd_{Post} \times 100}$$

where the Donor_post_ is the fluorescent value of CFP after bleaching and Donor_Pre_ is the fluorescent value of CFP prior to bleaching. BkGrd is the background fluorescence at the respective frame.

### Fura2-AM calcium imaging

Experiments were completed as previously described^40^. YFP-DAT cells were plated on glass bottom dishes and grown for 48–72 h. Cells were washed twice in HBSS (Life Technologies) followed by 30-min treatment in 5 μM Fura-2 AM (Life Technologies). Cells were then washed twice with HBSS followed by 30-min treatment with either vehicle or 1 μM PRE-084. Cells were then imaged on a Nikon Ti Eclipse inverted microscope. Fluorescence was monitored using dual excitation wavelengths (340/380 nm) and a single emission wavelength (510 nM). Fura-2 binds free calcium and excited at 340 nm, whereas unbound Fura-2 is excited at 380 nm. Baseline images were taken for 1 min followed by addition of vehicle, PRE-084 or METH. PRE-084 was added to a final concentration of 1 μM and METH at a final concentration of 10 μM. Images were acquired for 4 min after drug addition. To determine the DAT-dependence of the effect of METH, METH treatment experiments were performed in parental HEK cells not expressing DAT. For analysis, ROIs were detected on YFP-DAT images to encompass to entire internal region of the cell. The ratio of 340/380 was taken and normalized to the average fluorescence of the first 1 min of baseline imaging prior to drug treatment for each cell. The percent change in fluorescence over baseline was calculated as:

$$\% {\mathrm{\Delta }}F{\mathrm{/}}F_0 = \left( {F_{\rm {max}} - F_0} \right)/F_0 \times 100$$

where *F*_max_ is the maximal fluorescent value after METH addition and *F*_0_ is the average fluorescence at baseline. Images were presented as the ratio of 340/380 imaging.

### GCaMP6f calcium imaging

Cultured midbrain neurons were treated with AAV1-GCaMP6f day 4 in vitro. After 4–6 days, neurons were imaged on a Nikon Ti-E Inverted Live Cell Imaging System. GCaMP was measured at an excitation of 470 nm. For all conditions, baseline images were acquired for 30 s followed by 4 min and 30 s of imaging after treatment. For controls, external solution or 1 μM PRE-084 was added after baseline imaging. For METH treatment, neurons were incubated with 1 μM PRE-084 or vehicle for 30 min at 37 °C prior to imaging. After baseline imaging, 10 μM METH was added. To determine the selectivity of the dose of PRE-084, neurons pretreated with 1 μM BD1063 for 15 min prior to PRE-084 treatment. The DAT-dependence of METH-mediated increase in calcium was tested by pretreating the neurons with 10 μM nomifensine for 15 min prior to METH treatment. For quantification, experimenter-defined ROIs were created around the cell body of each individual neuron. Background fluorescence was subtracted from each frame. The percent change in fluorescence over baseline was calculated as described above. Images are presented in grayscale.

### Statistical analysis

Data analysis was performed via Prism 7 (GraphPad Software Inc.). Sample sizes were not determined by sample size calculations, but represent sample sizes similar to those generally used in the field for the respective types of experiments. Blinding was used to collect data. Statistical analyses were designed assuming normal distribution and similar variance among groups tested. One-way, two-way, or repeated measures ANOVA (with post-hoc tests as indicated for each experiment) were used for the statistical comparison of more than two means. The two-tailed, unpaired *t*-test was applied when two means were compared. Significance at *P* < 0.05 was considered statistically significant.

### Data availability

The data that support the findings of this study are available from the authors upon reasonable request.

## Electronic supplementary material

Supplementary Information

Description of Additional Supplementary Files

Supplementary Movie 1

Supplementary Movie 2
