# Supplementary material for: The sigma-1 receptor modulates methamphetamine dysregulation of dopamine neurotransmission
Source: Nat Commun. 2017 Dec 20;8:2228. doi: 10.1038/s41467-017-02087-x (PMC5738444; doi:10.1038/s41467-017-02087-x)
Supplement: Supplementary file 1 — Supplementary Information [file 41467_2017_2087_MOESM1_ESM.pdf]

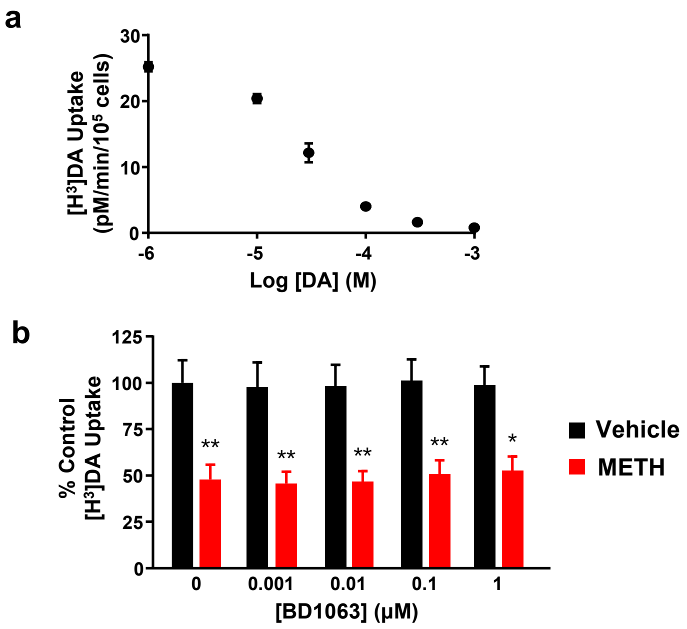

Supplementary Figure 1. Similar to σ_1R agonist treatment, σ_1R antagonist does not affect baseline or METH-inhibition of DA uptake. [^{3}H]DA uptake was measured in FLAG-DAT cells. **(a)** Competition curve for cold dopamine with 10 nM [^{3}H]DA. The concentration of cold dopamine at half maximum (3 μM) was used for all studies. **(b)** 10 μM METH (15 minutes) significantly decreased [^{3}H]DA uptake compared to vehicle control cells. Treatment with the σ_1R antagonist BD1063 had no effect on basal DA uptake or the METH-inhibition of uptake. Data is expressed as percent vehicle control (n = 3 independent experiments; $F_{(1,20)} = 67.42$, $P < 0.0001$, two way ANOVA Control vs METH; $F_{(4,20)} = 0.07587$, $P = 0.9888$, two way ANOVA for BD1063 treatment; Bonferroni's test for multiple comparisons of Vehicle vs. METH, *$P < 0.05$ and **$P < 0.01$). Data is represented as mean ± SEM.

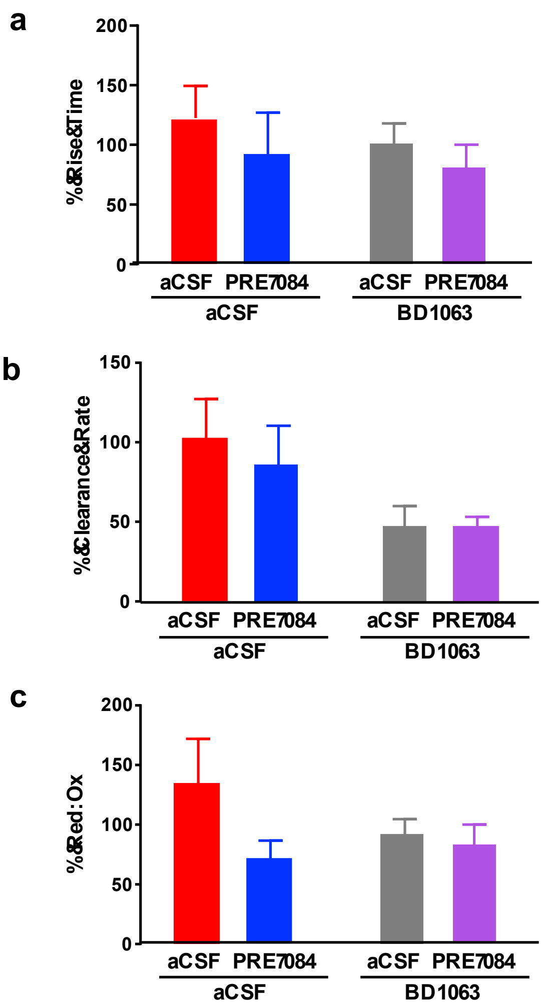

Supplementary Figure 2. Clearance profile of dopamine transmission in the dorsal striatum following agonist activation of σ_1R. PRE-084, BD1063, or combined treatment do not affect the **(a)** rise time, **(b)** clearance rate, or **(c)** reduction and oxidation ratio (Red:Ox) of METH-stimulated dopamine release in the striatum compared to animals receiving aCSF (n = 6 for aCSF + aCSF, n = 9 for aCSF + PRE-084, n = 4 for BD1063 + aCSF, n = 6 for BD1063 + PRE-084). The identity of neurotransmitter(s) released *in vivo* can be determined by the ratio of currents produced by reduction and oxidation (red:ox) of the neurotransmitter(s) at the carbon fiber recording electrode. *In vivo*, the ratio for dopamine (DA) is typically greater than 0.5, whereas the ratio for serotonin (5-HT) is typically less than 0.1. Ratios between 0.1 and 0.5 indicate a mix of DA and 5-HT [1]. While the dorsal striatum is predominantly innervated by DA terminals, it also receives appreciable serotoninergic innervation. In addition to evoking release of DA via DAT, METH also causes serotonin (5-HT) release via the serotonin transporter (SERT). Consistent with this, red:ox "signatures" for METH-induced neurotransmitter release ranged from 0.26 to 0.82, averaging 0.48 ± 0.07. This indicates that signals recorded at the carbon fiber electrode were a mix of DA and 5-HT. Interestingly, when analyzing group data, red:ox values were unchanged following PRE-084. If σ_1R regulation of the actions of METH were specific to DA neurons, then the expectation would be for red:ox values following PRE-084 to shift in favor of a 5-HT "signature" (i.e. moving closer to 0.1). The finding that red:ox values did not shift raises the possibility that σ_1Rs might similarly regulate serotonin transporter (SERT)-dependent 5-HT release following METH. Data is represented as mean ± SEM.

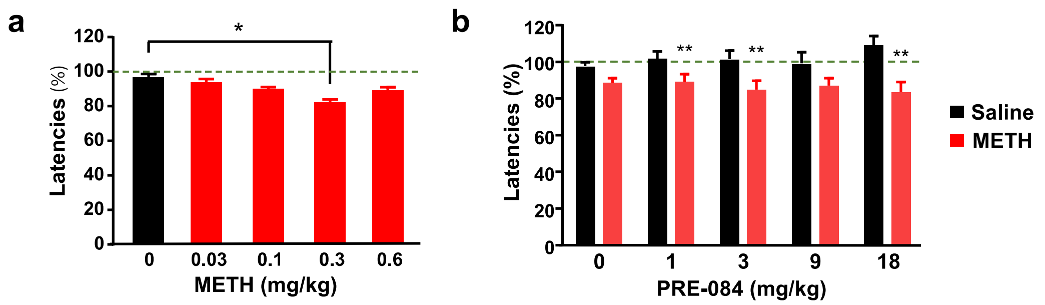

Supplementary Figure 3. METH influences the ICSS latency. (a) METH treatment decreased the ICSS response latency. Data is presented as percent control (no METH treatment) (*n* = 8 rats; $F_{(4,28)} = 3.08$, $P = 0.032$, one way ANOVA; Dunnett's test for multiple comparisons, $*P < 0.05$). **(b)** PRE-084 treatment does not affect ICSS latency or elevate the ICSS thresholds and therefore it is neither aversive nor produce negative mood state (*n* = 6 saline rats, 8 METH rats; $F_{(1,12)} = 15.31$, $P = 0.0021$; two way ANOVA; Bonferroni's test for multiple comparisons, $**P < 0.01$). Data is represented as mean ± SEM.

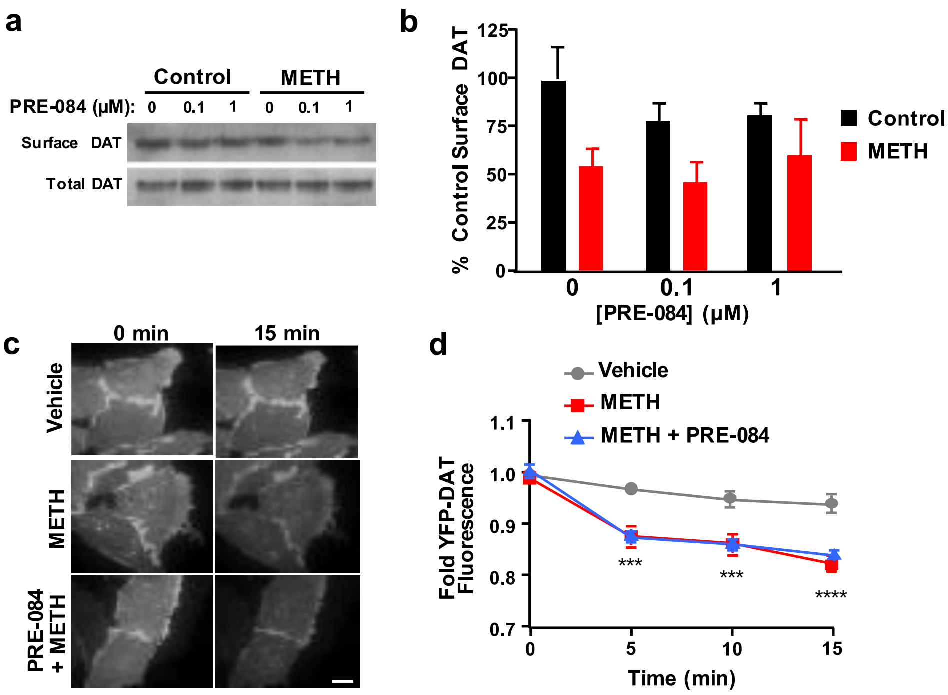

Supplementary Figure 4. σ_1R agonist does not influence METH-mediated DAT internalization. YFP-DAT cells were pretreated with 0.1 or 1 µM PRE-084 (30 min) followed by 10 µM METH or vehicle for 15 minutes. Cells were then treated with sulfo-NHS-SS-biotin to detect surface proteins. **(a)** Representative immunoblot of surface DAT and total DAT levels following drug treatment. **(b)** Bar graphs shows METH treatment decreased surface expression of DAT. Pretreatment with PRE-084 prior to METH exposure did not influence METH-mediated DAT trafficking (n = 3 independent experiments; $F_{(1,18)} = 9.029$, $P = 0.0076$, two way ANOVA, Control vs. METH). **(c)** Representative TIRF microscopy images of surface YFP-DAT following vehicle or 10 µM METH treatment over time in untreated or 1 µM PRE-084 (30 minutes) pretreated YFP-DAT cells. **(d)** Live cell TIRF microscopy was used to determine time-dependent cell surface redistribution of YFP-DAT. Line graph shows METH treatment (n = 21 cells) significantly reduced DAT levels at the membrane compared to vehicle treatment (n = 23 cells) as measured by a decrease in the fluorescent signal following up to 15 minutes of METH exposure. PRE-084 treatment (n = 18 cells) did not affect the constitutive or METH-induced YFP-DAT cell surface redistribution ($F_{(2,14)} = 13.07$, $P = 0.0006$ two way repeated measures ANOVA for Treatment, $F_{(3,42)} = 111$, $P < 0.0001$ two way repeated measures ANOVA for Time; Tukey's test for multiple comparisons, ***$P < 0.001$, ****$P < 0.0001$ for Vehicle vs. METH and Vehicle vs. METH + PRE-084). Data is represented as mean ± SEM.

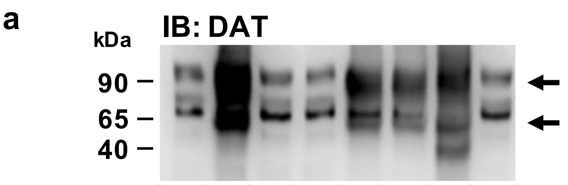

	1	2	3	4	5	6	7	8
Parental Cells	+			+				
DAT		$+^s$			$+^s$	$+^s$	+	+
σ_1R			$+^s$		+		$+^s$	
σ_1R EYFP				$+^s$		+		$+^s$

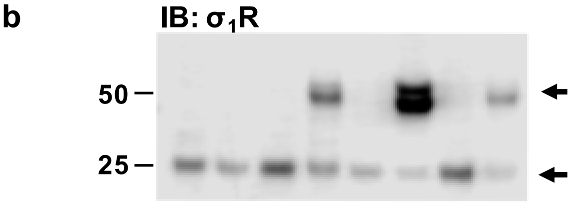

	1	2	3	4	5	6	7	8
Parental Cells	+			+				
DAT		$+^s$			$+^s$	$+^s$	+	+
σ_1R			$+^s$		+		$+^s$	
σ_1R EYFP				$+^s$		+		$+^s$

Supplementary Figure 5. DAT and σ_1R expression in crude lysates. Prior to IP, crude extracts of samples were analyzed by Western blot to verify expression in the samples. **(a)** DAT mature (upper arrow) and immature (lower arrow) were detected using mAB16 anti-DAT antibodies and whereas there are non-specific bands present in each lane, DAT specific bands are observed in samples expressing DAT (Lanes 2, 5, 6, and 7) **(b)** σ_1R (lower arrow) and σ_1R-EYFP (upper arrow) were detected using anti-σ_1R B-5 antibodies. The representative blots depict the findings from ≥ 3 independent experiments.

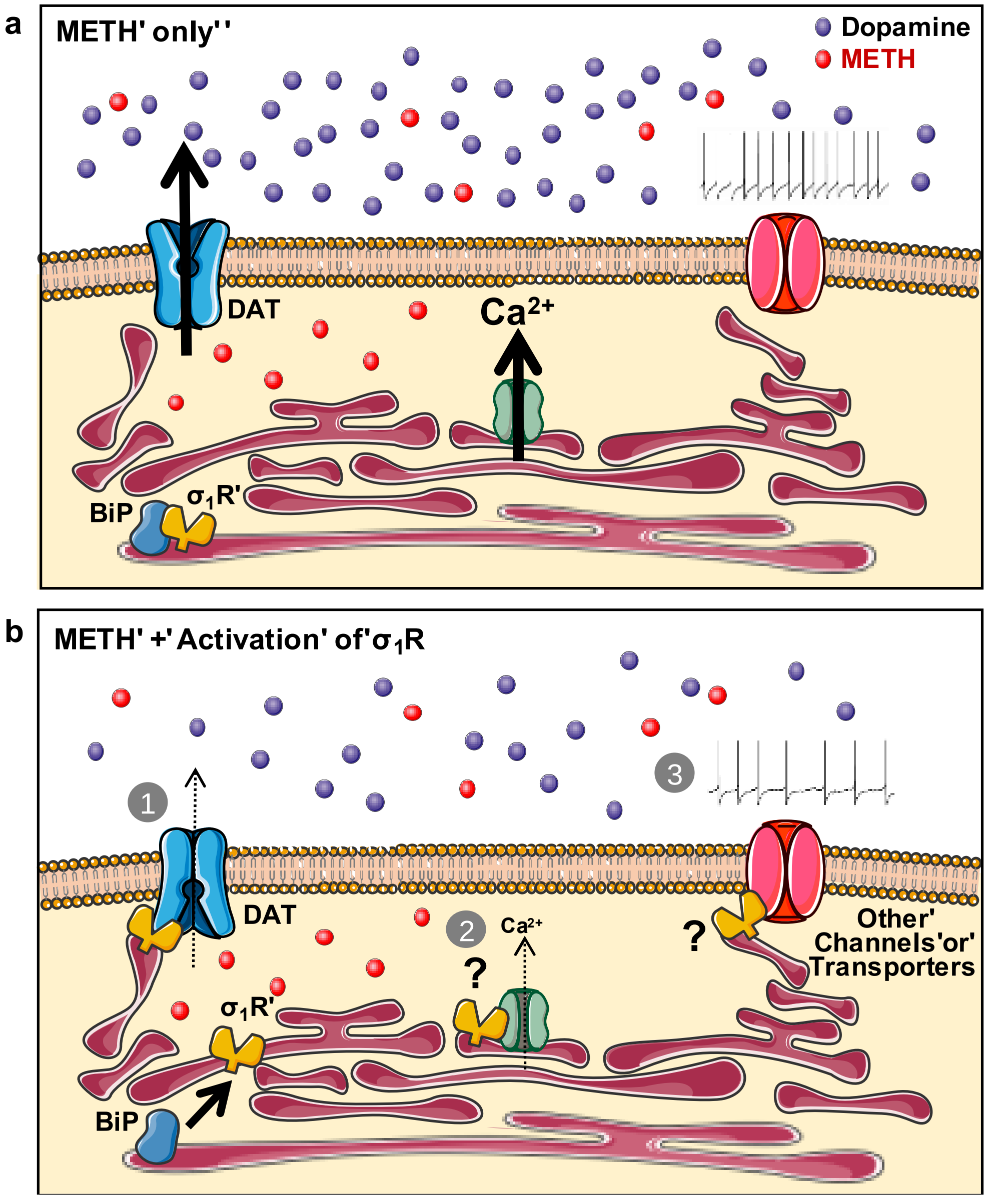

Supplementary Figure 6. σ_1R activation modulates METH-mediated cellular responses. (a) In control conditions, methamphetamine (METH) increases dopamine levels through interactions with the dopamine transporter (DAT). METH-stimulated reverse transport of dopamine via DAT occurs in a calcium (Ca^{2+})-dependent manner. METH also depolarizes the membrane resulting in increased firing activity. **(b)** σ_1R activation by PRE-084 in the presence of METH results in the dissociation of σ_1R from BiP such that σ_1R can then translocate within the cell and affect various cellular pathways to reduce METH-induced neurotransmission. Three potential molecular mechanisms are proposed that may be occurring independently or in combination. (1) σ_1R interacts with DAT at the plasma membrane, thereby decreasing METH-mediated dopamine efflux via DAT activity. This may occur via direct or indirect protein-protein interactions. (2) σ_1R decreases METH-stimulated increases in intracellular Ca^{2+}, leading to decreased METH-stimulated dopamine efflux. This can occur via modulation of intracellular Ca^{2+} stores, as shown, or interactions of σ_1R with Ca^{2+} channels. (3) Not investigated in this study but hypothesized to be a potential mechanism, σ_1R activation may also result in σ_1R-mediated interactions (direct or indirect) with other transporters or ion channels, particularly K^+ channels. This would promote hyperpolarization of the neuron and decrease METH-stimulated increases in firing activity. Cell membrane, molecules, proteins, and ER images are courtesy of Servier Medical Art, licensed under CC BY 3.0.

Supplementary Table 1. Antibodies

Name	Host Species	Source, Catalog #	Application	Concentration
DAT	Rat	Millipore, MAB369	IHC, biotinylation	1:1000
DAT	Mouse	Millipore, MABN669	coIP	1:40
Sigma-1 Receptor	Rabbit	Life Technologies, 42-3300	IHC, WB	1:500
Sigma-1 Receptor	Rabbit	Gift from Arnold Rhouho	coIP	1:40
Sigma-1 Receptor	Mouse	Santa Cruz, sc-137075	coIP	1:20
Tyrosine Hydroxylase	Rabbit	Millipore, AB152	IHC	1:1000
Green Fluorescent Protein	Mouse	Sigma Aldrich, G6539	WB	1:2000
Living Colors® A. V. (JL-8)	Rabbit	Clonetech, 632381	coIP	1:50
Living Colors® A. V. (JL-8)	Rabbit	Clonetech, 632381	WB	1:1000
Drosophila SERT	Rabbit	Alpha Diagnostics, AL-SERT13-A	coIP	1:50

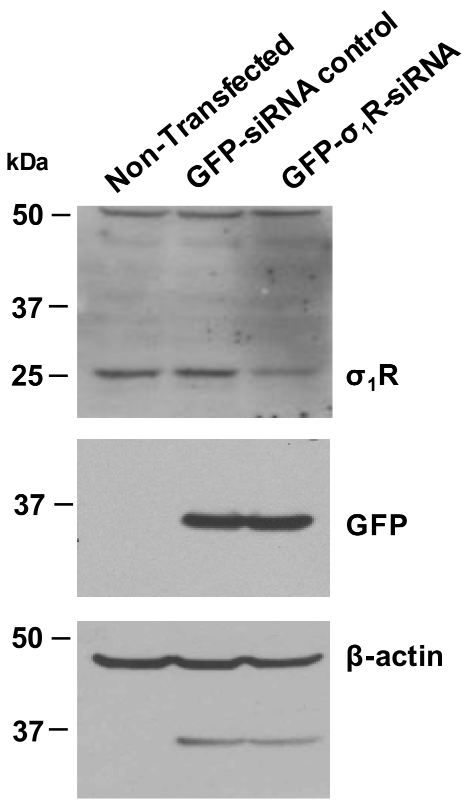

Supplementary Figure 7. Uncropped western images. Western blot images corresponding to images shown in Fig. 1f. Faint bands on the β-actin blot around 37 kDa represent residual signal from GFP.

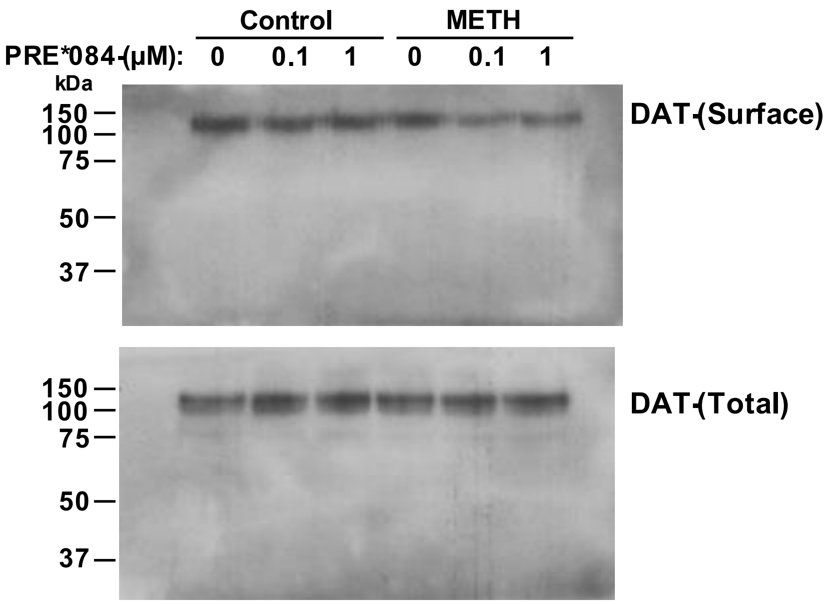

Supplementary Figure 8. Uncropped western images. Western blot images corresponding to images shown in Supplemental Fig. 4a. The molecular weight corresponds to the YFP-tagged DAT.

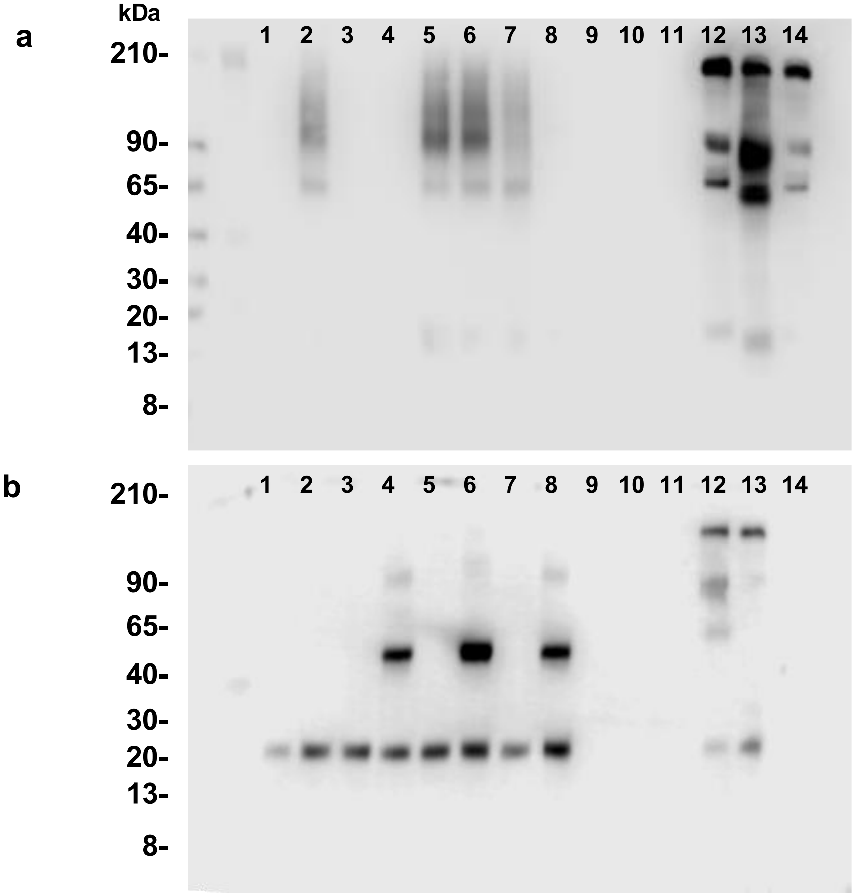

	1	2	3	4	5	6	7	8	9	10	11	12	13	14
Parental Cells	+													
DAT		$+^{s}$			$+^{s}$	$+^{s}$	+	+				$+^{s,n}$	$+^{s,n}$	$+^{s,n}$
σ_1R			$+^{s}$		+		$+^{s}$							
σ_1R-EYFP				$+^{s}$		+		$+^{s}$						
Control Resin									+					
No Antibody										+				
Unrelated Antibody											+			

Supplementary Figure 9. Uncropped western images. Western blot images corresponding to images shown in **(a)** Figure 8a and **(b)** Figure 8b. Lanes before lane 1 represent the molecular weight marker. Lanes 12 through 14 represent the same sample loaded at different concentrations of protein. Lane 14 is not represented in Figure 8 but is included here for completion.

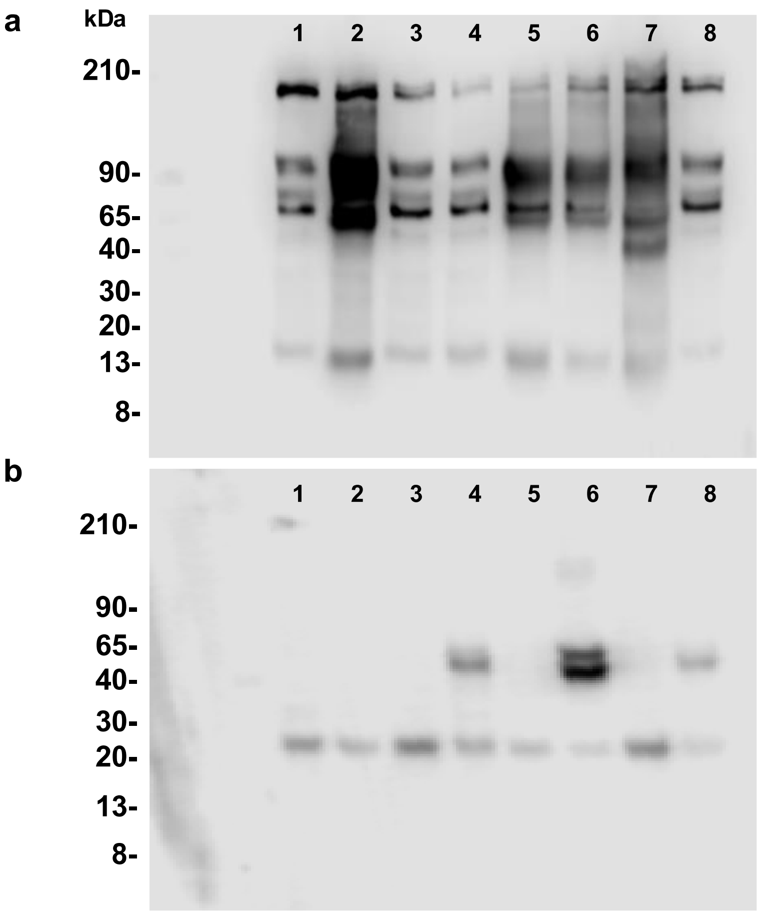

	1	2	3	4	5	6	7	8
Parental Cells	+			+				
DAT		+s			+s	+s	+	+
σ1R			+s		+		+s	
σ1R EYFP				+s		+		+s

Supplemental Figure 10. Uncropped western images. Western blot images corresponding to images shown in **(a)** Supplementary Fig. 5a and **(b)** Supplementary Fig. 8b.

1. Hoffman AF, Gerhardt GA. Differences in pharmacological properties of dopamine release between the substantia nigra and striatum: an in vivo electrochemical study. *The Journal of pharmacology and experimental therapeutics* **289**, 455-463 (1999).
