# Supplementary material for: The sigma-1 receptor modulates methamphetamine dysregulation of dopamine neurotransmission
Source: Nat Commun. 2017 Dec 20;8:2228. doi: 10.1038/s41467-017-02087-x (PMC5738444; doi:10.1038/s41467-017-02087-x)
Supplement: Supplementary file 2 — Description of Additional Supplementary Files [file 41467_2017_2087_MOESM2_ESM.pdf]

File Name: Supplementary Movie 1
Description: σ_1R dynamically interacts with DAT at the plasma membrane. Time-lapse images of YFP-DAT expressing cells revealed the dynamic movement of σ_1R-CFP located at ER/plasma membrane junctions as indicated by localization of YFP-DAT. These areas of interaction between σ_1R-CFP at the ER and YFP-DAT at the plasma membrane appear to occur at the previously described structures called cortical ER.

File Name: Supplementary Movie 2
Description: Activation of σ_1R attenuates the peak amplitude of METH-induced intracellular Ca^{2+} mobilization in GCaMP6f expressing neurons. Neurons were incubated with either vehicle or PRE-084 prior to treatment with METH and subsequent imaging; video begins immediately following METH addition. Lookup tables for both the in-color and corresponding intensity heat map are matched to compare absolute intensity, however, data reported is increase in fluorescence relative to baseline levels prior to drug addition (both AFUs) to account in intra-neuronal variability in baseline activity.
